# Phenotypic and functional patterns of murine bone marrow-derived dendritic cells influenced by *in vitro* differentiation conditions

**DOI:** 10.1371/journal.pone.0356806

**Published:** 2026-08-26

**Authors:** Irina Elena Ionescu, Catalin Tucureanu, Raluca Elena Chelmus, Vlad Constantin Tofan, Fabiola Margareta Ionita, Adrian Onu, Monica Neagu, Crina Stavaru, Iuliana Caras

**Affiliations:** 1 Department of Research and Development, “Cantacuzino” Institute, Bucharest, Romania; 2 Faculty of Biology, University of Bucharest, Bucharest, Romania; 3 “Titu Maiorescu” University, Faculty of Pharmacy, Bucharest, Romania; 4 Department of Immunology, “Victor Babes” National Institute, Bucharest, Romania; 5 Department of Pathology, Colentina Clinical Hospital, Bucharest, Romania; European Institute of Oncology, ITALY

## Abstract

The selection of an appropriate differentiation protocol for murine bone marrow-derived dendritic cells (BM-DCs) still presents a challenge due to the inherent plasticity of dendritic cells (DCs) and the high diversity of generation methods. As such, it is essential to evaluate *in vitro* BM-DCs characteristics during initial steps of experimental model development and tailor the protocols according to specific research goals while preserving the functional and phenotypic properties of DCs. In our study, we observed relevant variations in phenotypic and functional characteristics of BM-DCs differentiated with GM-CSF alone (GDCs) compared to those differentiated with GM-CSF and IL-4 (G4DCs). We found that GDCs exhibited high levels of costimulatory molecules, indicating a phenotypically mature state, but preserved the ability to respond to LPS (lipopolysaccharide) maturation stimuli by cytokine production. Moreover, cytokine secretion patterns observed in prime/ boost co-culture systems indicate the potential of Ag-pulsed BM-DCs to prime naive T cells. In contrast, while addition of IL-4 resulted in cells with a more immature phenotype that are responsive to LPS-driven maturation, it also promoted nonspecific T cell signaling that could interfere with *in vitro* evaluation of priming and antigen-specific T cell responses. These results highlight the impact of specific differentiation conditions that impose on the phenotypic and functional patterns of BM-DCs. Furthermore, the study emphasizes the significance of reassessing, even widely, the already employed protocols for DCs differentiation, and moreover, our study can provide valuable insights and guidance for novel *in vitro* experimental designs based on BM-DCs.

## Introduction

Over the last decades, dendritic cells have been a hallmark of both extensive and evolving research and clinical application, ranging from immune response studies to immunotherapy using dendritic cells (DCs)-based vaccination [1–3]. This large array of applications is due to DCs’s unique ability to initiate and shape antigen-specific immune responses as a result of continuously sampling their environment, engulfing foreign material, and presenting antigens to naive T cells. Their plasticity was exploited by the development of *in vitro* generated DCs and further extended to vaccine immunogenicity studies concluding to one of their clinical applications. Advancement in cellular ontogeny studies that triggered an extensive development of *in vitro* generated DCs in murine and human models [4–6] led to a considerable number of methods for cell differentiation. The implied methods for differentiating DCs from precursors have long-established protocols [7–9] that were developed in an array of methods tailored to different DCs subsets [10–14]. The extensive number of studies in which DCs were generated *in vitro* by altering the type of growth factors and their concentrations resulted in various differences in DCs phenotype and/or in their important functions, such as antigen processing and presentation. To further complicate matters, factors like specific timelines for DCs differentiation from progenitor cells, additional purification steps, age and strain of the animal from which progenitors were harvested, cell culture media components or even the type of culture plates, may determine subtle variations in DCs marker expression and may modulate their functions [15–17]. For instance, longer incubation periods and prolonged stimulus contact can lead to extensive maturation that can potentially affect antigen internalization or even generate so-called ‘exhausted’ DCs that can impact migration behavior or induce T cell polarization towards Th2 responses [18,19].

DCs are routinely generated *in vitro* following culture of murine bone marrow precursors (BM) or human monocyte precursors by using granulocyte-macrophage colony stimulating factor (GM-CSF) with or without interleukin-4 (IL-4) [8,20–22]. The main reason for *in vitro* production of DCs is the low number of cells that can be isolated *in vivo* from various tissues, making murine bone marrow-derived dendritic cells (BM-DCs) and DCs derived from human peripheral blood monocytes vastly used models. Unlike the human setting where IL-4 is considered essential for DC generation, murine BM-DCs are most often generated either by culturing with GM-CSF alone or in combination with IL-4 [23–25]. Phenotypically BM-DCs are CD11c + MHC class II+ cells that resemble tissue conventional DCs (cDCs) with different expression levels of costimulatory molecules, such as CD80, CD86 and CD40, depending on the activation state. Functionally, *in vitro* generated DCs respond to stimulation by microbial components through TLR (Toll-like receptor) signaling pathways with distinct patterns of cytokines/chemokines having pleiotropic effects on subsequent cells and overall immune responses [26]. Cytokine and chemokine profiles of resting immature or activated DCs are important markers in evaluating their functionality.

Previous research published more than 10 years ago has shown that GM-CSF promotes the differentiation of a heterogeneous mixed population that includes both functional DCs and macrophages with distinct marker expression based on ontogenetic, morphological, and gene expression criteria [20,27]. Additionally, *in vitro* studies focusing on DCs-based cancer vaccination have shown that the immature phenotype of DCs generated by GM-CSF alone is ideal for evaluating antigen and nanoparticulated vaccines uptake [28]. The use of IL-4 to *in vitro* differentiation protocols has led to multiple variations relevant to the immunological functions of BM-DCs. Addition of IL-4 can result in a better yield, increased expression in MHC class II (MHC II), in costimulatory molecules and significantly higher production of IL-12. *In vivo* immunotherapy studies using BM-DCs differentiated with various stimuli have suggested that an intermediate level of maturation, as observed with the addition of IL-4 to the protocol, is necessary to induce tumor-specific immune responses [29–31]. Contradictory results were published by other groups where IL-4 presence inhibited the differentiation of some bone marrow precursors into CD11c+ cells. Although it was shown that DCs generated in serum free media in the presence of IL-4 were phenotypically more mature and stronger stimulators of allogeneic splenocytes and antigen-specific T cells than DCs cultured in the absence of IL-4, both populations displayed a similar capacity to take up antigen and release IL-12 in response to maturation stimuli [32]. Taking on board the contradictory results regarding the specific *in vitro* conditions for differentiation in the present study we compared the phenotype and functional characteristics of BM-DCs generated using GM-CSF alone (GDCs) in comparison to the cellular model using GM-CSF with IL-4 (G4DCs). Besides the investigation of the induced phenotypes, in our experimental models we have evaluated also the functionality of the induced cells, namely the capacity of BM-DCs to prime naive T cells in an *in vitro* model consisting of a BM-DCs/naive lymphocyte co-culture system.

## Methods

### Animals and ethics approval

All experiments were performed in accordance with EU Directive 2010/63 for the protection of animals used for scientific purposes, the national and institutional guidelines for animal care and were approved by the Cantacuzino Institute Animal Ethics Committee, by the Ethics Committee of the University of Bucharest nr. 111/01.08.2025 and by National Authority ANSVSA number 06/24.02.2025. BALB/c male mice, bred by the Cantacuzino Institute’s animal husbandry, were maintained under specific pathogen-free (SPF) conditions. A total of 12 mice, age 6–8 weeks, were used in these experiments. Animals were euthanized by administration of an overdose of anesthetic consisting of Xylazine 2% and Ketamine 10%.

### Bone marrow cell isolation

BM mononuclear cells were isolated from the tibia and femur of BALB/c mice according to standard protocol [33]. Briefly, both ends of the epiphyses were excised and the medullary cavity was flushed out with PBS using a syringe. The bone marrow was gently dispersed by pipetting and filtered through a 70-µm strainer in order to remove small pieces of bone and tissue debris. The single cell suspension was centrifuged at 300 x g for 10 minutes and the cell pellet was resuspended in RPMI medium (Lonza-BioWhittaker, Basel, Switzerland) containing 10% FCS (Thermo Fisher Scientific, Waltham, USA), 2 mM L-glutamine (Lonza-BioWhittaker), 1% penicillin-streptomycin (Lonza-BioWhittaker) and supplemented with 50 µΜ 2-mercaptoethanol (Sigma-Aldrich, Saint Louis, USA). Cell counting and viability were performed by fluorescent staining with AO/PI (acridine orange/propidium iodide – BD Pharmingen™, San Jose, USA) using an inverted microscope (Nikon Eclipse TE2000-U with Nikon DS-Qi2 camera).

### Generation and culture of BM-DCs

BM-DCs were generated by culture with either GM-CSF (Recombinant mouse GM-CSF, Biolegend, San Diego, USA, cat. 576308) alone or in conjunction with IL-4 (Recombinant mouse IL-4, R&D Systems, Minneapolis, USA, cat. 404-ML-050), starting from a BM mononuclear cell suspension of 0.2 x 10^6^ cells/mL in 6-well plates. GM-CSF was added at a concentration of 20 ng/mL and IL-4 at a concentration of 20 ng/mL for the first stimulation. Every 3 days, a third of the volume was replaced with fresh media containing GM‐CSF (40 ng/mL) w/wo IL‐4 (10 ng/mL).

**Fig 1:**
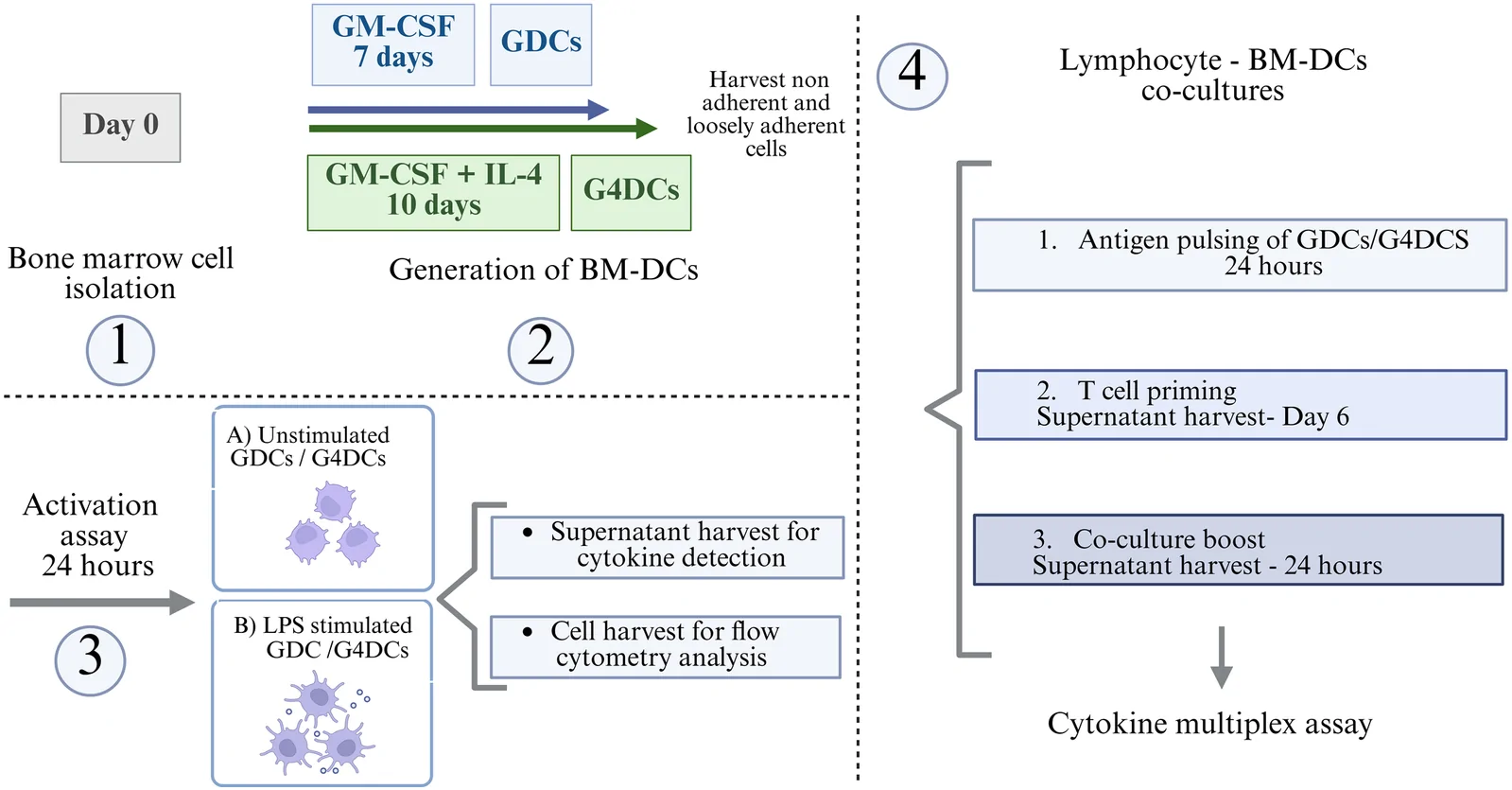
Experimental design for BM-DCs differentiation and phenotypic and functional analyses. Created in BioRender. Ionescu, I. (2026) https://BioRender.com/vrb0luq.

Non-adherent and loosely adherent cells were harvested by gently washing the well surface on day 7 for GDCs cultures and on day 10 for G4DCs cultures. Harvested GDCs and G4DCs cells were used in subsequent flow cytometry and cytokine detection analysis. The firmly adherent fraction was discarded. To assess the activation of BM-DCs, a cell suspension of 1 × 10^6^ cells/mL was transferred to a 24-well plate (Ultra low attachment, Corning, New York, USA) and was treated with 100 ng/mL LPS (TLR4 Agonist – Ultrapure LPS from *E. coli* 0111:B4, InvivoGen, San Diego, USA) which is commonly employed to induce DCs maturation. After 24 hours, the supernatant of unstimulated and LPS-stimulated cells was harvested for cytokine detection, while cells were used for phenotypic analysis. A schematic experimental design is presented in Fig 1.

### Cytokine detection

Supernatant of LPS-treated and untreated BM-DCs was tested for cytokine secretion. We selected a panel of 5 cytokines, IL-6, TNF-α, IL-10, IL-12p40, and IL-12p70, which are known to be expressed and secreted by mature DCs. Cytokines were measured using commercially available ELISA reagents, according to the manufacturer’s instructions (ELISA Duoset, R&D Systems).

### Cell surface molecules analysis

Cell surface expression of CD11c, MHC II, and CD86 were determined using a FACSCanto flow cytometer (BD FACSCanto II; BD Biosciences, San Jose, USA). Briefly, single-cell suspensions were washed in PBS containing FCS (2%), and Fc receptor activity was blocked by incubating cells with anti-CD16/CD32 antibody (TruStain FcX PLUS, BioLegend, cat. 156604) at 4°C for 15 minutes. Cell surface staining was performed using specific antibody mixes (CD11c - APC anti-mouse CD11c, BioLegend, cat. 117310, MHC II – FITC anti-mouse I-A/I-E, BioLegend, cat. 107606 and CD86 – PE anti-mouse CD86, BioLegend, cat. 105008) or corresponding isotype-matched control antibodies (BioLegend: APC Armenian Hamster IgG Isotype Ctrl, cat. 400912, FITC Rat IgG2b, κ Isotype Ctrl, cat. 400634, PE Rat IgG2a, κ Isotype Ctrl, cat. 400508). After incubation at 4°C for 30 min, cells were washed, resuspended in washing buffer and analyzed by flow cytometry. Prior to acquisition, an unstained cell suspension was mixed with 2.5 µg/mL of 4′,6-diamidino-2-phenylindole (DAPI) to exclude dead cells. Data was further analyzed with FlowJo software™ v.10.7.2 (BD Life Sciences). After debris and doublet exclusion, DAPI-negative viable cells were selected followed by gating of the CD11c⁺ population. MHC-II and CD86 expression was assessed within the CD11c⁺ compartment as percentages and geometric median fluorescence intensity values (GeoMFI) of CD11c positive cells or CD11c+MHCII + /CD86 + cells. The gating strategy is shown in S4 Fig.

### Antigen pulsing of BM-DCs

Immature GDCs and G4DCs cultures were transferred to 96-well round bottom tissue culture plates at 10^4^ cells per well and pulsed with 10 µg/mL of antigenic protein (Ag) for 24 hours. The antigenic protein used was a truncated form of influenza virus hemagglutinin A/Puerto Rico/8/1934(H1N1) developed in-house, namely the ectodomain expressed in a prokaryotic system and refolded [34]. Endotoxin testing of the antigen preparation was performed using a LAL Chromogenic Endpoint Assay (HycultBiotech, HIT302). Immature BM-DCs without antigen (non-pulsed BM-DCs) were used as controls for baseline response. After the incubation, supernatants were removed, fresh media was added and cells were used in co-culture experiments.

### Lymphocyte – BM-DCs co-cultures

Naive T cells were purified from splenocytes by magnetic cell sorting. Briefly, spleens from 8-week-old male BALB/c mice were harvested, the splenic capsules were punctured and cells were gently nudged out with a curved needle, declumped by pipetting and filtered through a 70 µm strainer to prepare single-cell suspensions. Red blood cells were lysed with ammonium chloride (155 mM, ThermoFisher Scientific, Waltham, USA) for 5 minutes on ice before neutralization and washing with PBS. T cells were isolated from this single-cell suspension by negative selection using the mouse Pan T Cell Isolation Kit II (Miltenyi Biotec, Bergisch Gladbach, Germany) according to the manufacturer’s instructions.

The isolated CD3 + T cells were co-cultured at 0.2 x 10^6^ cells per well with 0.01 x 10^6^ antigen-loaded BM-DCs or non-pulsed BM-DCs (BM-DCs:T cells ratio of 1:20). Murine recombinant IL-2 (5 ng/mL, BioLegend, cat. 575408) was added to the co-cultures as a growth factor to support *ex vivo* expansion of T cells [35]. Co-cultures were incubated for 6 days with a third of the media removed and replaced with fresh IL-2-supplemented media on day 3. On day 6 the supernatant was harvested and stored at −80°C for later evaluation. In order to boost antigen-specific responses, on day 6, new antigen-loaded or control BM-DCs were added to the co-culture for another 24 hours followed by supernatant collection and storage. Samples from prime and boost stimulations were analyzed using a Luminex-based bead multiplex assay (LXSAMS-10, R&D Systems) in order to evaluate a panel of 10 cytokines secreted by various subclasses of T helper cells (IFN-γ, IL-1 beta, IL-2, IL-4, IL-5, IL-6, IL-23p19, IL-13, TNF-α, IL-10).

### Statistical analysis

All data visualization and statistical analyses were performed using the R programming language version 4.6.1 used in RStudio version 2023.09.1 + 494 [36,37].

Distribution normality was assessed group-wise using the Shapiro–Wilk test. Where normality assumptions were met the parametric student’s T-test was used to assess statistically significant differences between groups. T-tests were performed with Welch-Satterthwaite approximation to the degrees of freedom where unequal variances were assumed in unpaired samples. Data that deviated from a normal distribution were compared using non-parametric Wilcoxon signed-rank test (for paired samples) or Wilcoxon rank-sum test (for unpaired). Measurements were considered paired for unstimulated versus LPS stimulated cells within the same experiment under the same differentiation conditions and unpaired when comparing GDCs to G4DCs. Differences in cytokine secretion were assessed with parametric tests and non-parametric tests were applied on flow cytometry data. In all tests, p values were adjusted for multiple comparisons using the Benjamini-Hochberg procedure. Adjusted p values below 0.05 were considered statistically significant. Cytokine concentrations from unstimulated and LPS activated cells, expressed in pg/mL, were log10-transformed and represented as box and whisker plots using the ggplot2 package [38].

Hierarchical clustering of data from BM-DCs/lymphocyte co-culture system was calculated automatically (euclidean clustering distance) using the ComplexHeatmap package [39].

## Results

### Yield and phenotype of BM-DCs generated under specific cell culture conditions

In order to generate an optimal purity of DCs, as assessed by expression of the CD11c, the cell harvest period for GDCs was 8 days and 11 days for G4DCs. Adjustment of the harvest period was based on our previous experiments. Specifically, we observed that when G4DCs were cultured for 7–8 days, the percentage of CD11c+ cells was lower, often falling below 70% of total cells. However, by providing additional growth factors and extending the culture period for 3 more days, we were able to increase the percentage of CD11c+ cells to well above 80% (S1A Fig). Extending GDCs culture to 10–11 days did not increase the percentage of CD11c⁺ cells but further elevated basal MHC-II and CD86 expression. Therefore, GDCs were cultured for 7–8 days (S1B Fig). The total cell yield was found to be higher when DCs were differentiated in the presence of IL-4 (0.3 x 10^6^ ± 0.06 cells/ mL G4DCs in comparison to 0.13 x 10^6^ ± 0.05 cells/ mL GDCs).

**Fig 2:**
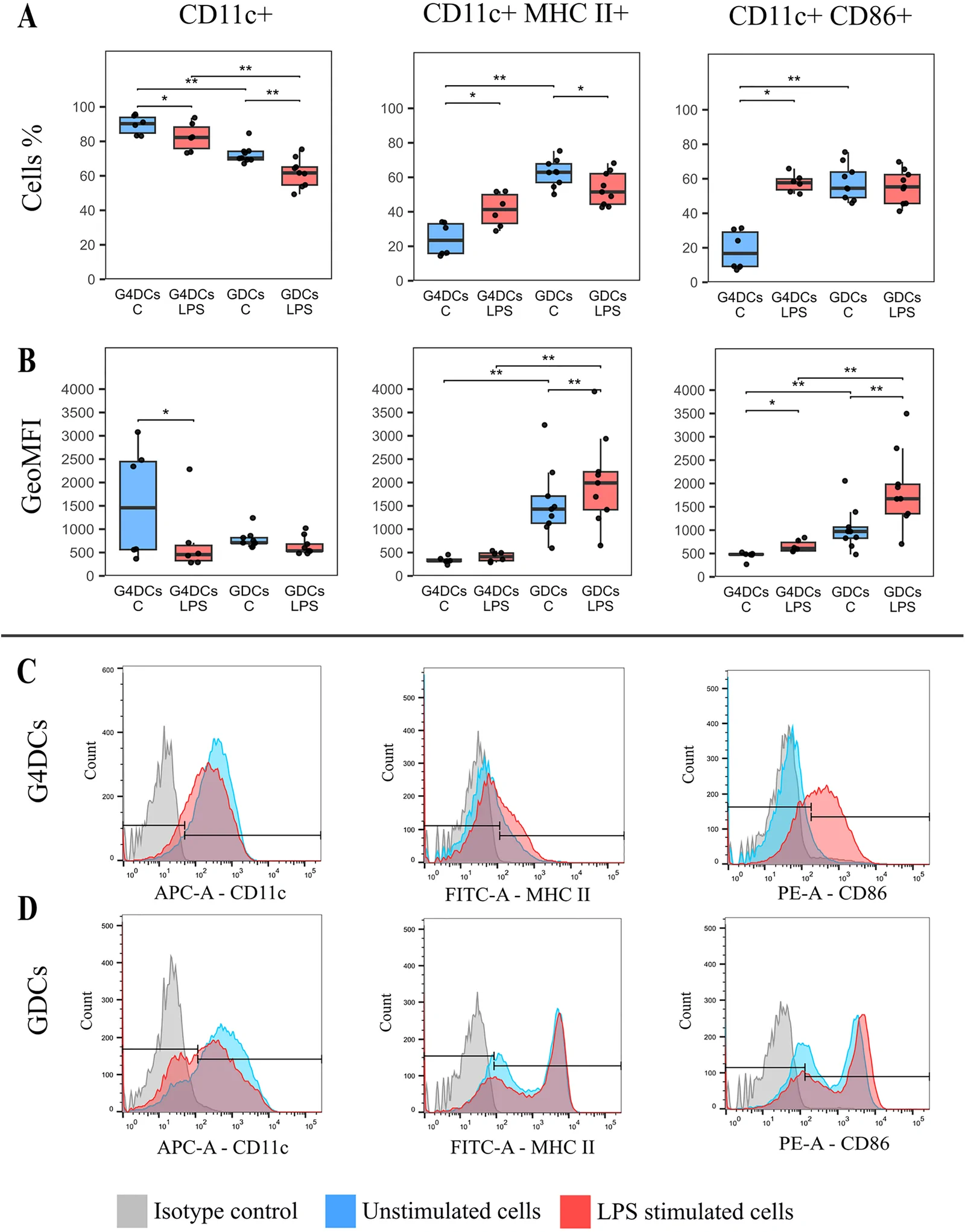
LPS maturation of BM-DCs generated with GM-CSF and IL-4 (G4DCs) or GM-CSF alone (GDCs). (A) Percentage of CD11c + , CD11c+MHC II+ and CD11c+CD86 + positive cells in unstimulated (C) or LPS-stimulated BM-DCs (LPS). (B) Geometric mean fluorescence intensity (GeoMFI) of CD11c + , CD11c+MHC II+ and CD11c+CD86 + positive cells. (C) Representative overlaid histograms showing marker expression in unstimulated and LPS-stimulated G4DCs, and (D) representative overlaid histograms showing marker expression in unstimulated and LPS-stimulated GDCs. Data was aggregated from 6 independent experiments for G4DCs cells (n = 6) and 9 independent experiments for GDCs cells (n = 9). * p < 0.05, ** p < 0.01, Wilcoxon signed-rank test (paired) for unstimulated vs. LPS-stimulated under the same differentiation conditions and Wilcoxon rank-sum test (unpaired) for G4DCs vs GDCs.

Maturation of BM-DCs following LPS stimulation was assessed by measuring the increase in surface expression of MHC II and CD86 compared to immature BM-DCs. Immature G4DCs showed a higher percentage of CD11c+ cells than GDCs. Upon LPS stimulation, both G4DCs and GDCs displayed a slight decrease of CD11c+ cells (Fig 2A). Immature GDCs cultures exhibited high percentages of MHC II+ and CD86 + cells and no significant increase in the percentage of these two surface markers was observed in response to LPS stimulation (Fig 2A). However, the LPS-induced response in GDCs was reflected by increased marker expression intensity, as shown by GeoMFI analysis (Fig 2B). In contrast, G4DCs cultures showed lower percentages of MHC II+ and CD86 + cells that significantly increased following LPS stimulation (Fig 2A and 2B). Representative flow cytometry histograms are shown in Fig 2C for G4DCs cell populations and in Fig 2D for GDCs cell population. Surface marker expression data in tabular form is presented in the supplementary section (S1 Table).

### Cytokine profile of GDCs and G4DCs cultures

**Fig 3:**
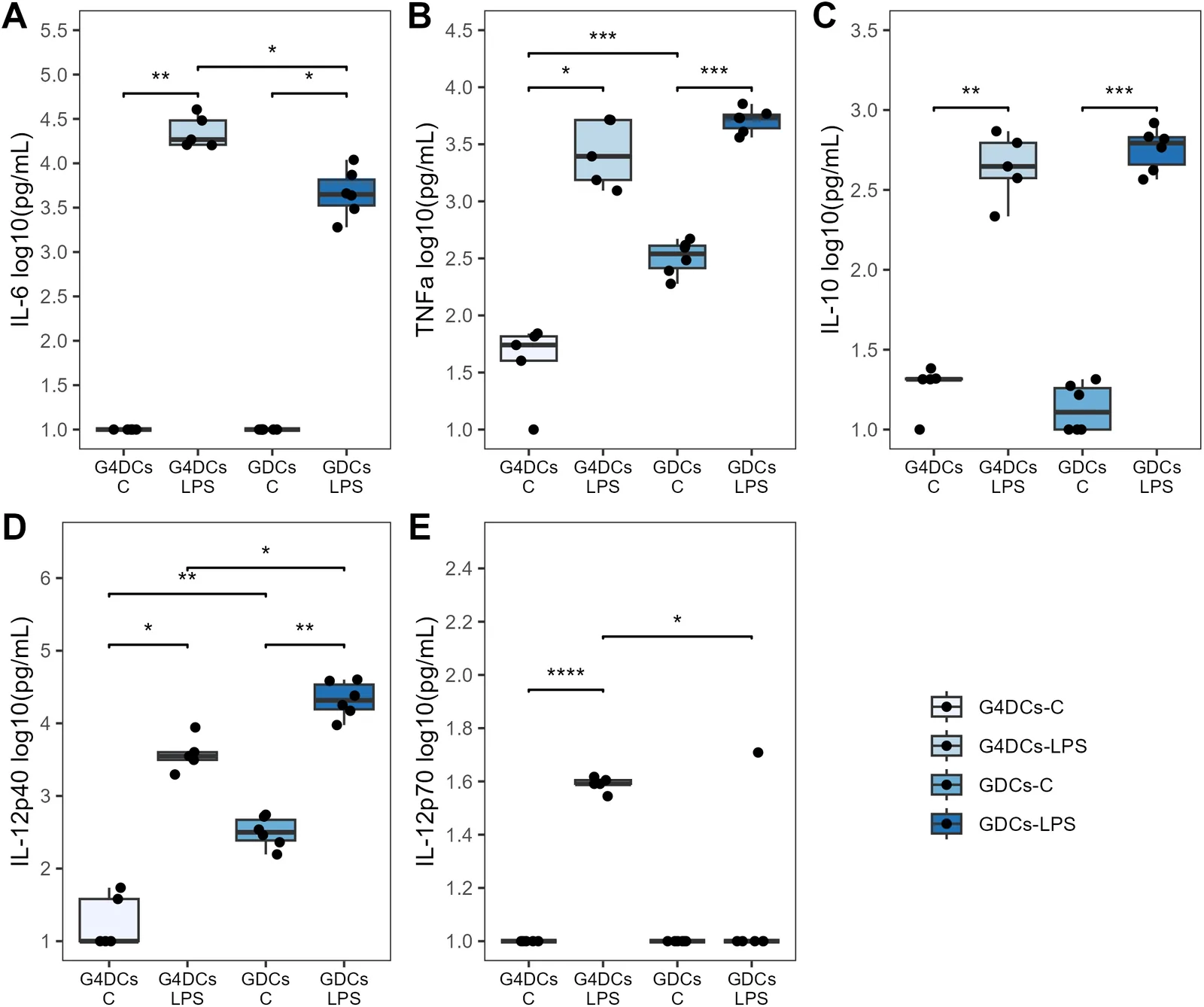
Cytokine secretion by control immature (C) and LPS-stimulated (LPS) BM-DCs generated with GM-CSF and IL-4 (G4DCs) or with GM-CSF alone (GDCs). (A) IL-6, (B) TNF-α, (C) IL-10, (D) IL-12p40 and (E) IL-12p70 concentrations in the culture supernatants were expressed as pg/mL and represented as boxplots and individual measurements (points) on a log10 scale. Data aggregated from 5 independent experiments for G4DC cells (n = 5) and 6 independent experiments for GDC cells (n = 6). Statistical analysis was performed on the non-transformed data using the paired t-tests to compare unstimulated and LPS-stimulated cells within the G4DC and GDC groups and unpaired t-tests to compare G4DCs with GDCs under unstimulated and LPS-stimulated conditions (* p < 0.05, ** p < 0.01, *** p < 0.001, **** p < 0.0001).

Activated DCs can produce a variety of polarizing cytokines that control adaptive immune responses. As the secreted cytokines by DCs vary according to the different developmental stages and the type of stimulation, a panel of 5 relevant inflammatory and Th-promoting cytokines (IL-6, TNF-α, IL-10, IL-12p40 and IL-12p70) was selected in preliminary experiments to assess cytokine secretion in immature and LPS-activated BM-DCs. IL-6 was not detectable in immature BM-DCs regardless of the generation protocol. Treatment with LPS induced a marked increase in IL-6 production in both differentiation conditions, but with significantly higher levels in G4DCs cultures compared with GDCs ones (Fig 3A). In contrast, immature GDCs secreted higher levels of TNF-α compared to immature G4DCs. Following LPS stimulation, TNF-α levels were significantly increased as compared to immature cells, but no significant difference was noted between the two generation protocols (Fig 3B). IL-10 was present in small amounts in both culture conditions and stimulation with LPS significantly increased production to similar levels (Fig 3C). Immature G4DCs secreted marginal levels of the IL-12p40 subunit as opposed to immature GDCs. IL-12p40 cytokine levels considerably increased upon stimulation in both conditions, with significantly higher levels for GDCs compared to G4DCs (Fig 3D). IL-12p70 heterodimer was marginally detected only in stimulated cell cultures belonging to the G4DCs group (Fig 3E).

### *In vitro* priming of naive T cells by antigen preloaded BM-DCs

To evaluate the ability of GDCs or G4DCs to initiate a primary immune response, naive T cells were exposed to Ag-pulsed BM-DCs and cytokine concentrations were measured in the cell culture supernatants. *In vitro* Ag-specific responses were boosted 6 days later by adding fresh Ag-pulsed BM-DCs to the cocultures. As the priming of naive T cells depends on DCs maturation state, in preliminary experiments we tested if the recombinant HA protein used for BM-DCs pulsing was by itself able to induce cell maturation. In accordance with previous reports [40], we noticed that the Ag used in our system induces BM-DCs maturation as evidenced by increased expression of MHC II and CD86 molecules in a similar fashion to LPS maturation (S2 Table). Endotoxin testing of the antigen preparation was performed in order to rule out any confounding effects due to potential LPS contamination, and the results showed below 121 EU endotoxin per mg of protein resulting in below 1.2 EU/mL in the BM-DC priming conditions.

**Fig 4:**
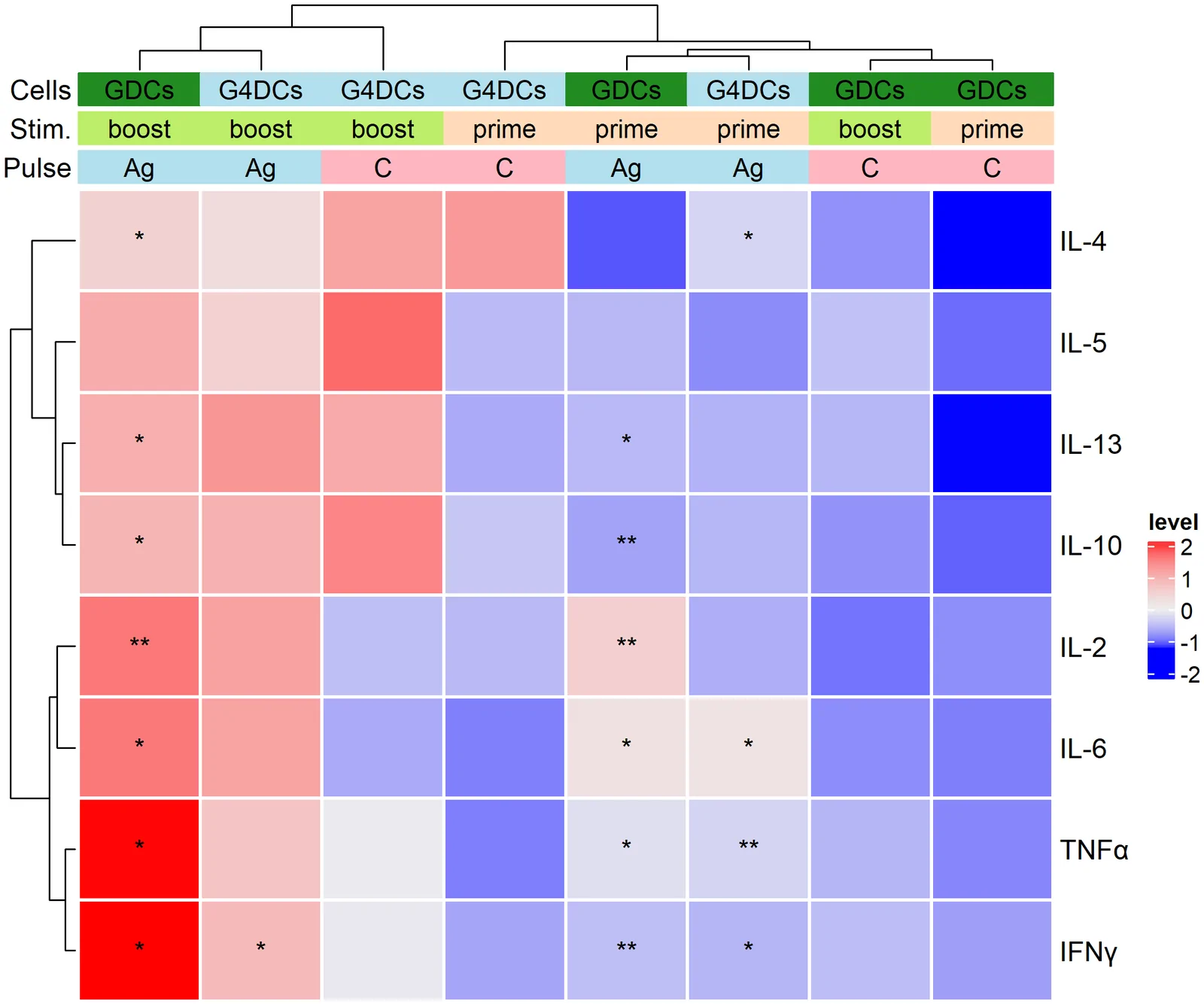
Heatmap and dendrograms representation of cytokine secretion in BM-DCs/ T cells *in vitro* co-cultures. Experimental conditions are defined by prime and boost stimulation of naive T cells with BM-DCs generated with GM-CSF alone (GDCs) or with GM-CSF and IL-4 (G4DCs) pulsed with antigen (Ag) or unpulsed (C). Cytokine concentrations are scaled and centered by row. The color key used is red:white:blue for high:average:low values on a log scale. Statistical significance is only presented for antigen to control comparisons in prime or boost for either GDCs or G4DCs (* p < 0.05, ** p < 0.01, unpaired t-tests). Complete analysis of intergroup differences is presented in S2 Fig.

Hierarchical cluster analysis of cytokine secretion in co-cultures of T cells with Ag-pulsed BM-DCs (dendrograms and heatmaps in Fig 4) allowed the identification of two main groups of cytokines differentiating culture and stimulation conditions. Priming of naive T cells with Ag-pulsed GDCs induced increased secretion of most of the analyzed cytokines. In particular, IL-13, IL-10, IL-2, IL-6, TNF-α and IFN-γ were significantly increased in Ag-pulsed versus unpulsed GDCs. For IL-4 and IL-5, an increased secretion was also observed compared to the control group, although not statistically significant. In addition, the magnitude of response was significantly increased for all cytokines tested in an Ag-dependent manner after the boost stimulation with Ag-pulsed GDCs. A mixed Th1 (IFN-γ, IL-2, TNF-α, IL-6) and Th2 (IL-4, IL-5, IL-10, and IL-13) cytokine response can be observed for naive T cells in both prime and boost stimulation with Ag-pulsed GDCs.

Priming of naive T cells with Ag-pulsed G4DCs induced a clear Ag-dependent Th1 response, with significant increases of IFN-γ, TNF-α and IL-6 levels compared to control G4DC stimulation. Additionally, IL-2 levels also increased in an Ag-dependent manner. However, IL-5, IL-10, and IL-13 levels remained comparable between the control and the Ag-pulsed groups and IL-4 level was significantly elevated in the control group compared to the Ag-pulsed group. Boost stimulation with Ag-pulsed G4DCs led to a further increase in response in the Th1 cytokines cluster (IFN-γ, IL-2, TNF-α, IL-6). Although IL-13, IL-5, IL-10 and IL-4 levels were higher in the boost co-cultures this was irrespective of antigen pulsing of the G4DCs indicating non-specific Th2 type background activation of these cells.

## Discussion

While BM-DCs have become an invaluable cellular resource for deciphering and detailing the different stages of the immune response, the multiple experimental protocols available to generate BM-DCs from bone marrow precursors lead to rather different cell populations regarding both phenotypical and maturation marker expression and key functionality. Therefore, it is becoming highly challenging to identify a reproducible method to perform BM-DC differentiation in a manner suitable for specific experimental designs in different research areas or at least to understand the limitations of current protocols when used in different experimental settings. Moreover, the diversity of the implied protocols makes difficult the comparison between the pathways developed within different labs and the obtained results. This issue is more so important when the experimental pathways of differentiation are supposed to led to clinical implementation. Since there are conflicting data regarding the benefit of IL-4 for generation of murine BM-DCs we have used GM-CSF alone or together with IL-4 to generate BM-DCs and we compared the phenotype and functional properties of these generated cells.

By optimizing the culture period, we were able to achieve similar percentages of CD11c positive cells in both culture conditions. Extending the duration of G4DC culture to 11 days allowed us to reach >80% CD11c⁺ purity, whereas shorter periods of 6–8 days as reported in other studies were suboptimal, with CD11c⁺ purity often below 60% [28,32]. This enabled us to more accurately compare BM-DCs generated from both protocols and to minimize the introduction of additional variables, such as the need for labeling and magnetic separation, which could potentially induce mechanical stresses and trigger spontaneous maturation of DCs [41,42]. In our hands, the use of GDCs resulted in high percentages of cells expressing maturation markers MHC II and CD86. In contrast, G4DCs showed low basal expression of MHC II and CD86 markers, in contrast with other studies where in certain conditions IL-4 was found to induce a more mature phenotype [43,44]. However, one study [27] indicated that the high expression of MHC II molecules in GDCs cultures may not be exclusively correlated with the spontaneous maturation status of DCs. This study demonstrated a heterogeneity in CD11c+MHC II+ population and showed that some features of DCs maturation might apply to certain types of macrophages [27]. Using a similar protocol for generating GDCs, we observed a comparable distribution of subpopulations characterized by high and low-intermediate expression of maturation markers (S3 Fig). However, one of the limitations of our study is that due to the low number of markers used in our panel for phenotypic evaluation, we are unable to fully attribute the high expression of maturation molecules to the heterogeneous populations presented in the GDCs.

Despite the contrasting phenotypes of BM-DCs derived from the two culture conditions, the cytokine profile of unstimulated cells and the enhanced response after LPS stimulation resembled features of functional DCs.

IL-12 secretion by DCs is critical in Th1 response development, while autocrine IL-10 production contributes to the balance and steering of Th1/Th2 responses by regulating IL-12 production [45]. We evaluated both the heterodimer IL-12 (IL-12p70) and the free IL-12p40 subunit. Previous studies have shown that the free IL-12p40 subunit can be secreted in significantly higher amounts compared to the heterodimer after exposure to a bacterial TLR ligand [46,47]. Additionally, it has been shown that DCs produce IL-12p70 only transiently, further secretion being dependent on CD40 ligation by CD154 expressed on activated CD4 + T cells [48]. This could account for the marginal levels of detection we observed for the heterodimer in BM-DCs cultures regardless of the presence of IL-4. In unstimulated GDCs cultures, the increased expression of maturation markers and relatively high secretion of IL12p-40 and TNF-α may indicate an activated state. However, upon stimulation with LPS, there was a marked increase in cytokine production, suggesting a robust activation of DCs in response to bacterial stimuli.

Preclinical evaluation of vaccine candidates relies on the capacity to prime T cells *in vitro* and the actual methods evaluating the priming capacity have important value in this endeavor. To date, there are several attempts in which human moDCs were successfully used to test for vaccine and adjuvant immunogenicity [49,50]. However, up to our knowledge, there are no currently available assays based on murine BM-DCs to prime naive T cells, in order to investigate vaccine immunogenicity *in vitro*. Here, we additionally investigated the ability of BM-DCs generated in the two different conditions and pulsed with influenza hemagglutinin as a model of viral Ag to prime naive T cells in an *in vitro* co-culture system.

Priming of naive T cells with Ag-pulsed GDCs induced a mixed Th1/Th2 cytokine response. This indicates that even though GDCs exhibited mature characteristics prior to exposure to stimuli, they were still capable of efficiently capturing antigens, processing and presenting the specific peptide in MHC II restriction in order to prime naive T cells. Furthermore, the functional capacity of GDCs to initiate a proper immune response in this *in vitro* setting is further supported by the increase in both Th1 and Th2 cytokines following boost stimulation of co-cultures with antigen-pulsed GDCs, suggesting subsequent expansion of antigen specific T cells. We can postulate that the mixed Th1/Th2 cytokine production is due to the core set of genes that are commonly induced following activation of murine DC subsets [51].

Despite exhibiting expected immature phenotypic characteristics and an enhancement of maturation markers after LPS or Ag activation when cultured alone (S1 Table), antigen primed G4DCs induced primarily Th1 type cytokine secretion in T-cell co-cultures, while Th2 type cytokines were elevated in boost cultures irrespective of antigen priming. Although DCs are not regarded as an important source of IL-4 [52], there is evidence suggesting that the addition of IL-4 in the generation protocol may induce production of Th2-polarizing cytokines within DCs cultures. However, there have been some discrepancies observed between the expression of IL-4 mRNA, intracellular detection and the actual secretion of IL-4 protein [53]. Other studies have found a direct correlation between the presence of IL-4 during DCs maturation and the production of IL-4 in T cells activated by these DCs using *in vitro* culture systems and *in vivo* studies [54,55]. As IL-4 promotes the differentiation of naive CD4 + T cells into IL-4 – secreting Th2 cells, the initial presence of IL-4 in co-culture system could lead to the nonspecific activation of T cells rather than T-cell receptor (TCR) engagement, particularly in the absence of an appropriate antigenic signal. Our data indicate that the two steps in experimental design, namely the generation of BM-DCs and the evaluation of BM-DCs/naive T cells response, are interdependent and must be tested side by side, in order to select the most appropriate approach depending on expected outcomes. However, further investigations and adjustments are required to develop a robust *in vitro* method for screening vaccine antigens or formulations via induction of primary murine T cell responses. Also, as our study is limited to cells derived from BALB/c mice, further optimization will probably be required when using a different murine model.

Based on our results and previous studies, the *in vitro* differentiation of BM-DCs and their APC functionality, depends on the cytokines and growth factors supplemented in cell culture or secreted by the cells, modulating the ability to prime naive T cells. Our results indicate the importance of the experimental design for *in vitro* assays based on BM-DCs for further studies or immunogenicity evaluation.

## Supporting information

S1 Fig Phenotypic analysis of G4DCs at different time points. Histograms showing gated CD11c+ cells (APC - CD11c+), MHC II+ cells (FITC – MHC II+) and CD86 + cells (PE - CD86+) and percentages of positive cells from (A) G4DCs cells and (B) GDCs cells, cultured for 7 days (blue color) and 10 days (red color).
(PDF)

S2 Fig Cytokine secretion in BM-DCs - T cells *in vitro* co-cultures. Experimental conditions were defined by prime and boost stimulation of naive T cells with BM-DCs generated with GM-CSF alone (GDCs) or with GM-CSF and IL-4 (G4DCs), either pulsed with antigen (Ag) or unpulsed (C). Cytokine concentrations (pg/mL) after prime (green) or boost stimulation (purple) are shown as boxplots. Statistical significance for comparisons between all groups in prime and boost conditions is indicated (* p < 0.05, ** p < 0.01; unpaired t-test).
(PDF)

S3 Fig High and low-intermediate expression of maturation markers in GDCs. Representative plots of CD11c⁺MHC II⁺ and CD11c⁺CD86 ⁺ cell populations from GDCs. Unstimulated cells (red plots) and LPS-stimulated cells (blue plots) were subdivided into subpopulations based on high or low-intermediate marker expression.
(PDF)

S4 Fig Bone marrow derived dendritic cells gating strategy. Debris were excluded based on FSC-A and SSC-A, followed by singlet selection and gating of DAPI-negative viable cells. Corresponding isotype-matched control antibodies were used to define baseline fluorescence. CD11c⁺ cells were subsequently identified from the viable cells and MHC-II and CD86 expression was evaluated within the CD11c⁺ population as the percentage of positive cells and geometric median fluorescence intensity (GeoMFI). At least 10,000 events from the DAPI-negative viable-cell gate were analyzed per sample.
(PDF)

S1 Table LPS maturation of BM-DCs generated with GM-CSF alone (GDCs) or GM-CSF and IL-4 (G4DCs). Percentages (%) and fluorescence intensity (GeoMFI) of CD11c + , populations are extracted from histograms and from double positive gating CD11c+MHC II+ and CD11c+CD86 + . Cell percentages and GeoMFI are represented as the mean + standard deviation (±) from 9 individual experiments for GDCs and from 6 experiments for G4DCs.
(DOCX)

S2 Table The percentages and expression levels for GDCs and G4DCs. Unstimulated cells and cells stimulated with LPS were used as controls to evaluate marker expression after stimulation with the antigen (HA_18–528_) prior to the co-culture system. Specific marker percentages and expression levels (gMean) were extracted from histograms.
(DOCX)
