# Supplementary material for: Phenotypic and functional patterns of murine bone marrow-derived dendritic cells influenced by in vitro differentiation conditions
Source: PLoS One. 2026 Aug 26;21(8):e0356806. doi: 10.1371/journal.pone.0356806 (PMC13513930; doi:10.1371/journal.pone.0356806)
Supplement: Supplementary file 1 [file pone.0356806.s001.pdf]

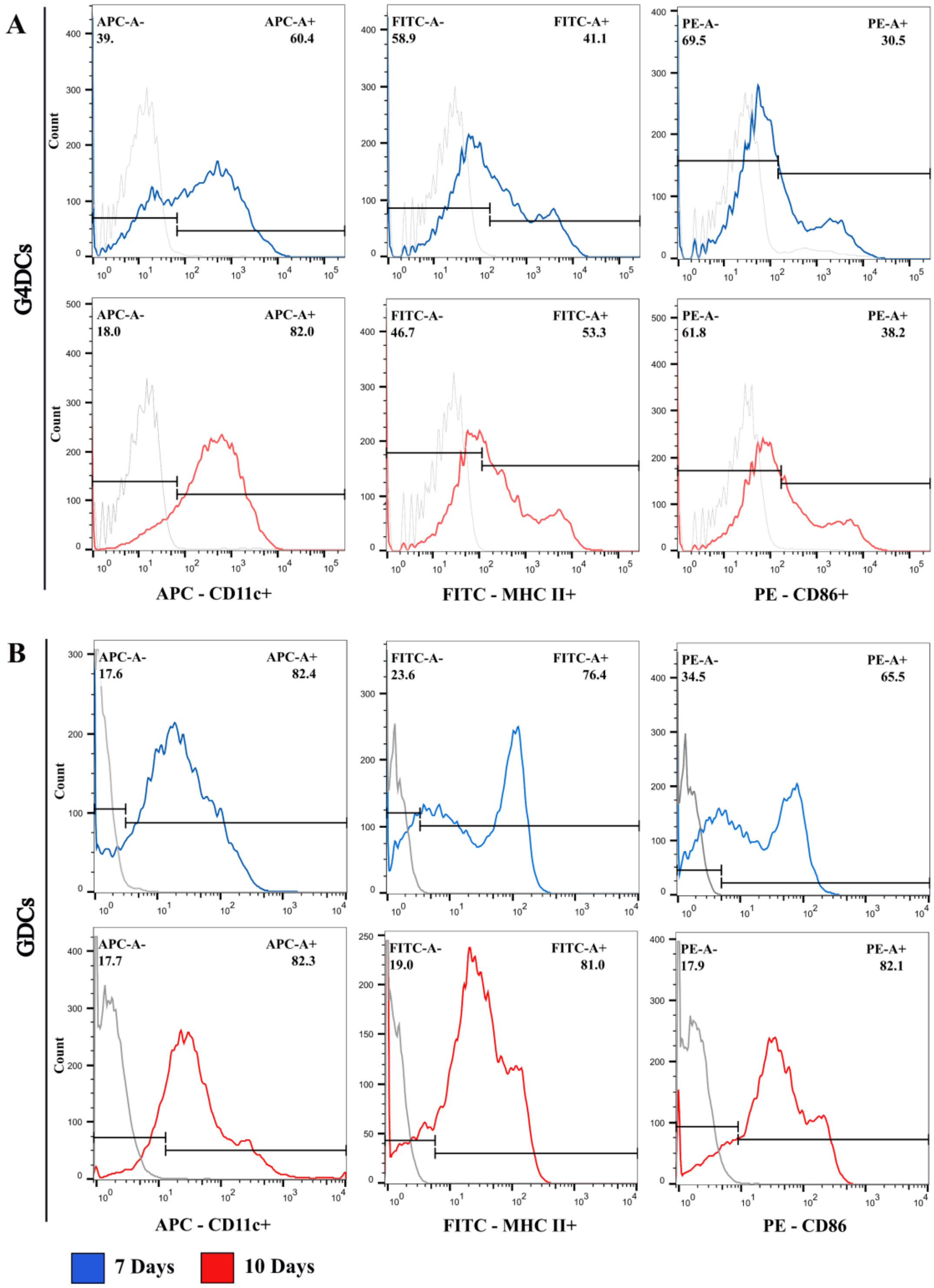

S1 Fig. Phenotypic analysis of BM-DCs at different time points.

Histograms showing gated CD11c+ cells (APC - CD11c+), MHC II+ cells (FITC - MHC II+) and CD86+ cells (PE - CD86+) and percentages of positive cells from (A) G4DCs cells and (B) GDCs cells, cultured for 7 days (blue color) and 10 days (red color).
