# Supplementary material for: Phenotypic and functional patterns of murine bone marrow-derived dendritic cells influenced by in vitro differentiation conditions
Source: PLoS One. 2026 Aug 26;21(8):e0356806. doi: 10.1371/journal.pone.0356806 (PMC13513930; doi:10.1371/journal.pone.0356806)
Supplement: Supplementary file 2 [file pone.0356806.s002.pdf]

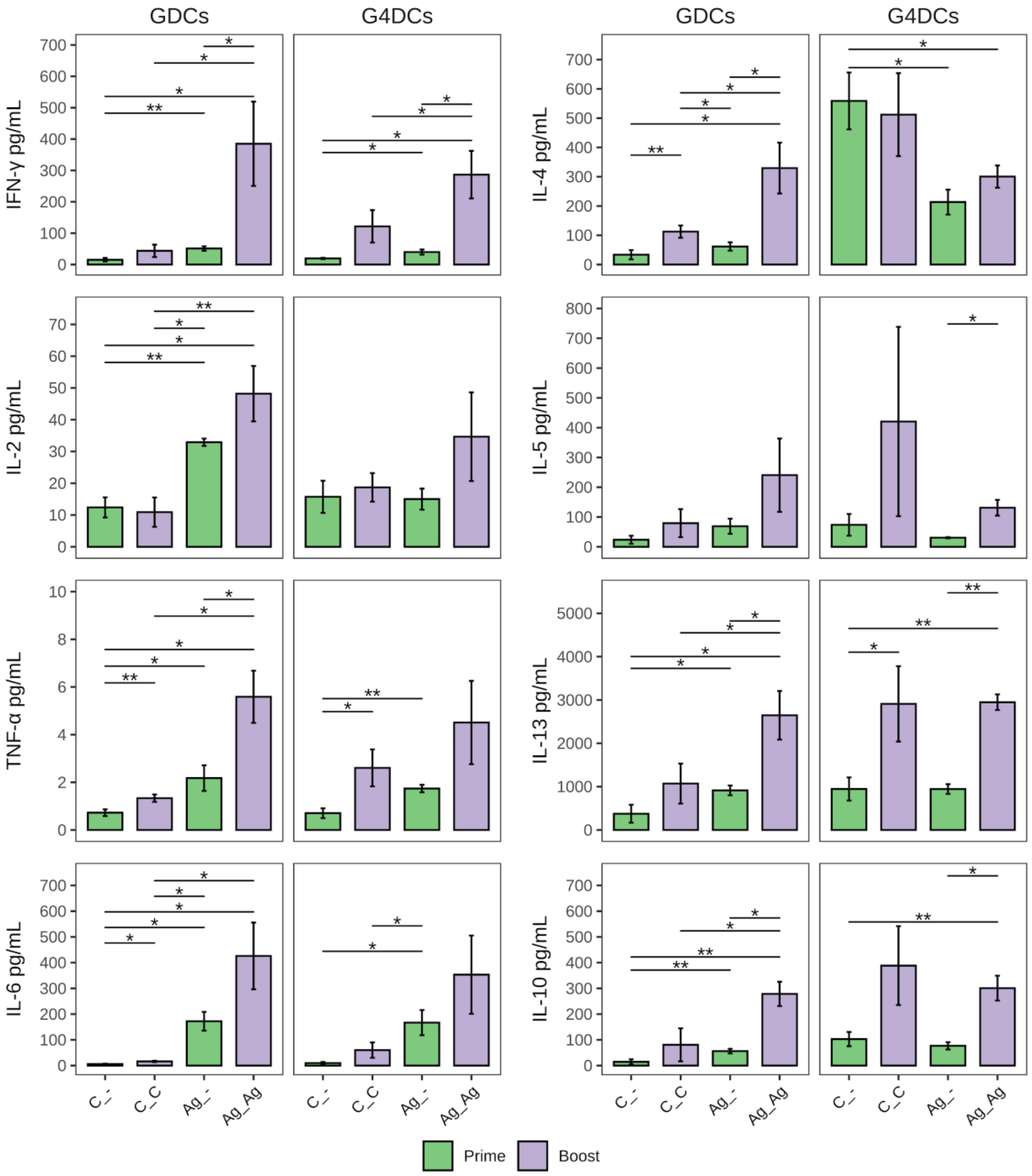

S2 Fig. Cytokine secretion in BM-DCs - T cells *in vitro* co-cultures.

Experimental conditions were defined by prime and boost stimulation of naive T cells with BM-DCs generated with GM-CSF alone (GDCs) or with GM-CSF and IL-4 (G4DCs), either pulsed with antigen (Ag) or unpulsed (C). Cytokine concentrations (pg/mL) after prime (green) or boost stimulation (purple) are shown as boxplots. Statistical significance for comparisons between all groups in prime and boost conditions is indicated (* $p < 0.05$, ** $p < 0.01$; unpaired t-test).
