# Supplementary material for: Phenotypic and functional patterns of murine bone marrow-derived dendritic cells influenced by in vitro differentiation conditions
Source: PLoS One. 2026 Aug 26;21(8):e0356806. doi: 10.1371/journal.pone.0356806 (PMC13513930; doi:10.1371/journal.pone.0356806)
Supplement: Supplementary file 3 [file pone.0356806.s003.pdf]

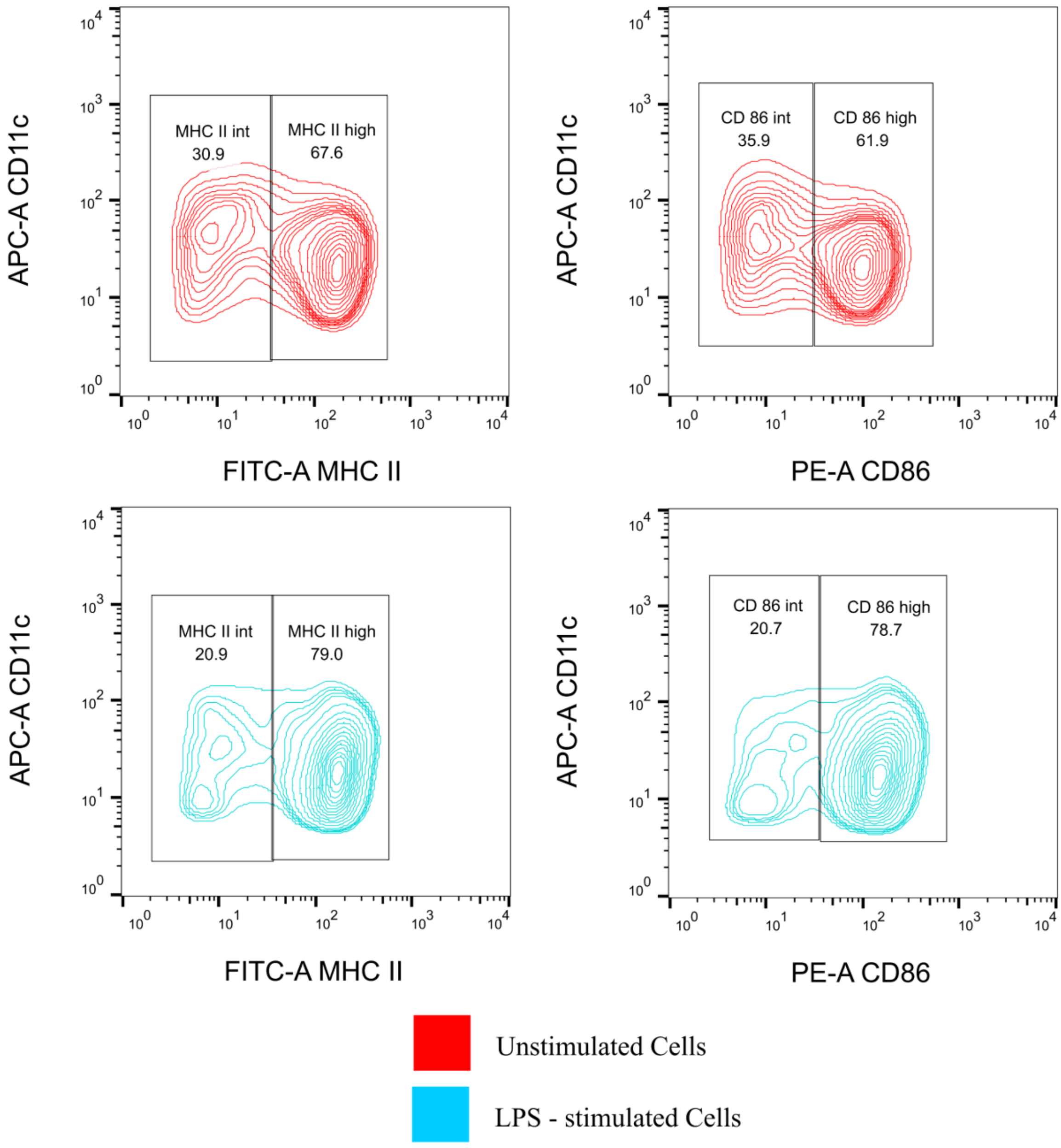

S3 Fig. High and low-intermediate expression of maturation markers in GDCs. Representative plots of CD11c⁺MHC II⁺ and CD11c⁺CD86⁺ cell populations from GDCs. Unstimulated cells (red plots) and LPS-stimulated cells (blue plots) were subdivided into subpopulations based on high or low-intermediate marker expression.
