# Supplementary material for: Phenotypic and functional patterns of murine bone marrow-derived dendritic cells influenced by in vitro differentiation conditions
Source: PLoS One. 2026 Aug 26;21(8):e0356806. doi: 10.1371/journal.pone.0356806 (PMC13513930; doi:10.1371/journal.pone.0356806)
Supplement: Supplementary file 4 [file pone.0356806.s004.pdf]

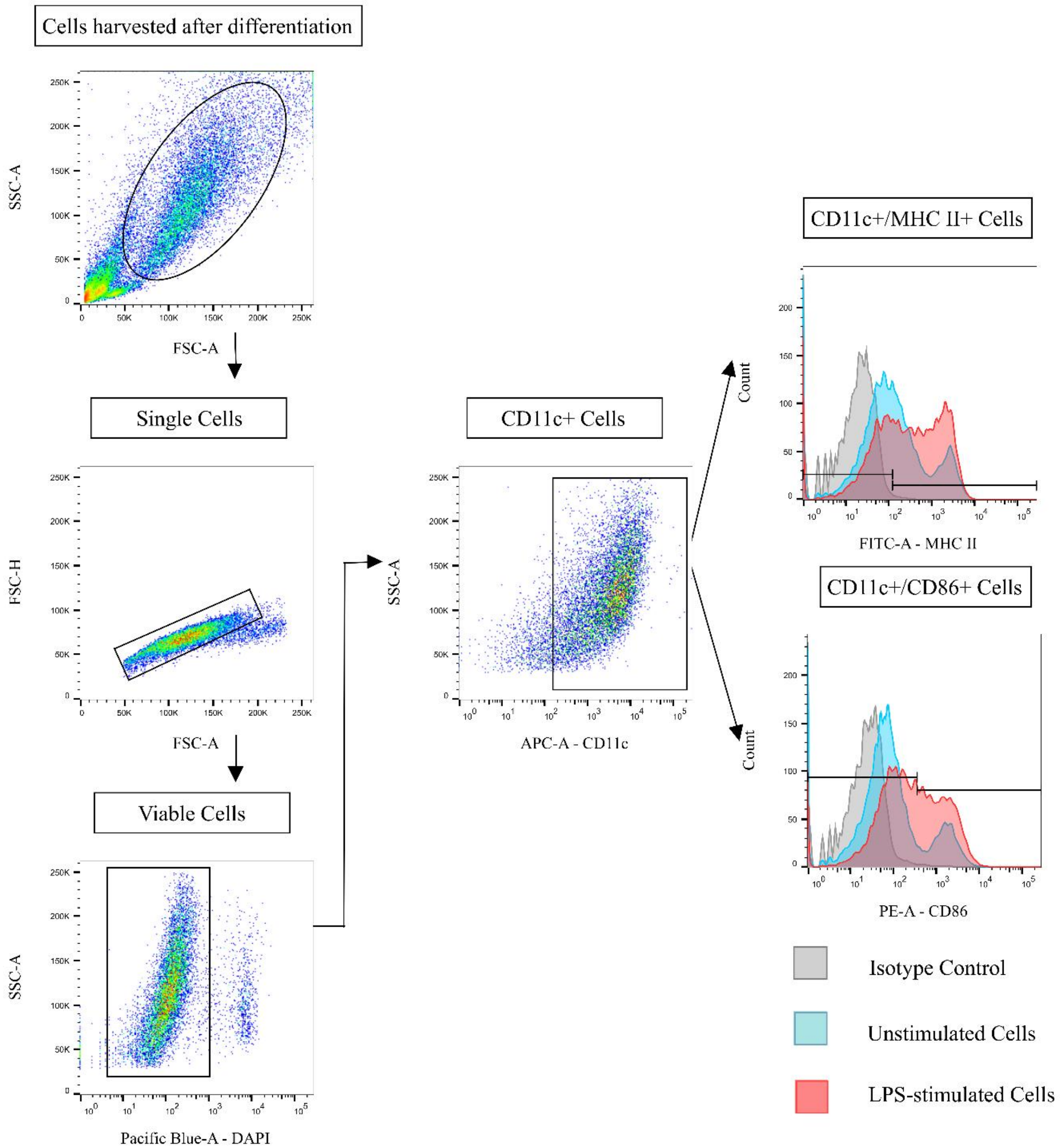

S4 Fig. Bone marrow derived dendritic cells gating strategy.

Debris were excluded based on FSC-A and SSC-A, followed by singlet selection and gating of DAPI-negative viable cells. Corresponding isotype-matched control antibodies were used to define baseline fluorescence. $CD11c^+$ cells were subsequently identified from the viable cells and MHC-II and CD86 expression was evaluated within the $CD11c^+$ population as the percentage of positive cells and geometric median fluorescence intensity (GeoMFI). At least 10,000 events from the DAPI-negative viable-cell gate were analyzed per sample.
