# Supplementary material for: Phenotypic and functional patterns of murine bone marrow-derived dendritic cells influenced by in vitro differentiation conditions
Source: PLoS One. 2026 Aug 26;21(8):e0356806. doi: 10.1371/journal.pone.0356806 (PMC13513930; doi:10.1371/journal.pone.0356806)
Supplement: Supplementary file 5 [file pone.0356806.s005.docx]

| Cell % | GDCs | | G4DCs | |
| --- | --- | --- | --- | --- |
|  | unstimulated | LPS | unstimulated | LPS |
| CD11c+ | 72.51 ± 5.2 | 61.92 ± 8.36 | 89.57 ± 5.45 | 82.58 ± 8.31 |
| % Cells from double positive gating | | | | |
| CD11c+MHC II+ | 62.59 ± 7.85 | 53.33 ± 9.64 | 24.17 ± 9.62 | 41.13 ± 9.88 |
| CD11c+CD86+ | 58.07 ± 10.49 | 55.53 ± 9.8 | 18.63 ± 11.38 | 57.57 ± 5.38 |
|  | | | | |
| GeoMFI | GDCs | | G4DCs | |
|  | unstimulated | LPS | unstimulated | LPS |
| CD11c+ | 780.88 ±  179.19 | 640.11 ±  180.95 | 1567.5 ± 1093.22 | 746.66 ± 155.34 |
| Cells from double positive gating | | | | |
| CD11c+MHC II+ | 1569.55 ±  724.27 | 2030 ± 918.55 | 335.83 ± 65.98 | 411 ±  94.67 |
| CD11c+CD86+ | 1030 ±  435.24 | 1873.33 ± 778.76 | 453.66 ± 84.36 | 653.66 ± 114.06 |

**S1 Table. LPS maturation of BM-DCs generated with GM-CSF alone (GDCs) or GM-CSF and IL-4 (G4DCs)**.

Percentages (%) and fluorescence intensity (GeoMFI) of CD11c+ populations are extracted from histograms and from double positive gating CD11c+MHC II+ and CD11c+CD86+. Cell percentages and GeoMFI are represented as the mean + standard deviation (±) from 9 individual experiments for GDCs and from 6 experiments for G4DCs.
