# Supplementary material for: Phenotypic and functional patterns of murine bone marrow-derived dendritic cells influenced by in vitro differentiation conditions
Source: PLoS One. 2026 Aug 26;21(8):e0356806. doi: 10.1371/journal.pone.0356806 (PMC13513930; doi:10.1371/journal.pone.0356806)
Supplement: Supplementary file 6 [file pone.0356806.s006.docx]

| Cell % | GDCs | | | G4DCs | | |
| --- | --- | --- | --- | --- | --- | --- |
|  | unstimulated | LPS | HA_18-528_ | unstimulated | LPS | HA_18-528_ |
| CD11c+ | 95.1 | 89.4 | 90.2 | 89.4 | 88.3 | 89.3 |
| MHC II+ | 86.9 | 90.2 | 86.7 | 37.6 | 57.5 | 61.3 |
| CD86+ | 84.9 | 86.7 | 92.6 | 29 | 57.2 | 63.2 |
| gMEAN | | | | | | |
| CD11c+ | 698 | 566 | 556 | 2569 | 2013 | 1933 |
| MHC II+ | 1391 | 2145 | 2327 | 719 | 930 | 995 |
| CD86+ | 1085 | 2604 | 2680 | 821 | 815 | 866 |

**S2 Table. The percentages and expression levels for GDCs and G4DCs.** Unstimulated cells and cells stimulated with LPS were used as controls to evaluate marker expression after stimulation with the antigen (HA_18-528_) prior to the co-culture system. Specific marker percentages and expression levels (gMean) were extracted from histograms.
