# Supplementary material for: Phenotypic and functional patterns of murine bone marrow-derived dendritic cells influenced by in vitro differentiation conditions
Source: PLoS One. 2026 Aug 26;21(8):e0356806. doi: 10.1371/journal.pone.0356806 (PMC13513930; doi:10.1371/journal.pone.0356806)
Supplement: Supplementary file 7 [file pone.0356806.s008.docx]

**Response to Reviewers**

Journal Requirements:

When submitting your revision, we need you to address these additional requirements.

1.Please ensure that your manuscript meets PLOS ONE's style requirements, including those for file naming. The PLOS ONE style templates can be found at

<https://journals.plos.org/plosone/s/file?id=wjVg/PLOSOne_formatting_sample_main_body.pdf> and

[https://journals.plos.org/plosone/s/file?id=ba62/PLOSOne_formatting_sample_title_authors_affiliations.pdf](https://track.editorialmanager.com/CL0/https:%2F%2Fjournals.plos.org%2Fplosone%2Fs%2Ffile%3Fid=ba62%2FPLOSOne_formatting_sample_title_authors_affiliations.pdf/1/010f019f16265b14-61055862-1354-4b1e-8dad-afefd00ea052-000000/7cwQQZwjv8Xr_FuYm9jbzkh8b2sOutjF9MAzXWuFOb4=258)

**Response:** We have carefully reviewed the PLOS ONE formatting and style requirements, including the recommendations for file naming. The revised manuscript and all accompanying files have been formatted and named accordingly, following the provided PLOS ONE templates.

2. Thank you for stating the following financial disclosure:

“This work was supported by the Ministry of Research and Innovation, Romania, PCCDI - UEFISCDI, project number PN-III-P1-1.2-PCCDI-2017-0529, Contract 62PCCDI/2018 (acronym CONVAC) and through the Core Program within the National Research, Development and Innovation Plan 2022-2027, with the support of National Research Authority (ANC), project no. 23 44.”

Please state what role the funders took in the study. If the funders had no role, please state: "The funders had no role in study design, data collection and analysis, decision to publish, or preparation of the manuscript."

If this statement is not correct you must amend it as needed.

Please include this amended Role of Funder statement in your cover letter; we will change the online submission form on your behalf.

**Response:** We confirm that the funders had no role in the study. The following statement has been added to the cover letter:

“The funders had no role in study design, data collection and analysis, decision to publish, or preparation of the manuscript.”

3. If the reviewer comments include a recommendation to cite specific previously published works, please review and evaluate these publications to determine whether they are relevant and should be cited. There is no requirement to cite these works unless the editor has indicated otherwise.

**Response:** We reviewed the publications recommended by the reviewers and evaluated their relevance to the scope and findings of our study.

**Reviewer #1**:

Dear authors,
The manuscript is interesting, and the obtained results show the impact of specific differentiation conditions on the phenotypic and functional patterns of BM-DCs.

**Response:** We thank the reviewer for their thoughtful comments and suggestions. We have revised the manuscript accordingly and hope that the changes adequately address all the points raised.

Because a new figure has been added, all subsequent figures have been renumbered accordingly. The line numbers cited in this response refer to the revised manuscript file labeled “Manuscript”.

1. The Introduction section is well written, but the Results section is quite confusing. I suggest drawing an experimental design for better comprehension by the reader in the Generation and culture of BM-DCs, Methodology section.

**Response:** We added to the **Methods** section, **Generation and culture of BM-DCs** subsection, a drawing of the experimental design. The figure is marked in the manuscript in **lines:** **149 -150** and in figure 1 legend **lines: 152-153**  “**Fig 1. Experimental design for BM-DCs differentiation and phenotypic and functional analyses**. Created in BioRender. Ionescu, I. (2026) [https://BioRender.com/vrb0luq](https://biorender.com/vrb0luq)” as required for licence compatibility between BioRender and PlosOne.

2. Line 133: How many viable cells do you have conducting this procedure? I strongly suggest to include for other research.

**Response:** For each BM-DCs batch, bone marrow mononuclear cells were obtained from one or more animals, depending on the requirements of the experiment and whether cells were also needed for additional experiments. The mean total cell yield was 42.8 ± 9.7 × 10⁶ cells per animal, with a viability of 92.8 ± 3.2%, corresponding to approximately 39.7 × 10⁶ viable cells per animal. Because the number of 6-well plates used varied among experiments, the total number of cells seeded per experiment also varied. Therefore to avoid confusion we reported the standardized initial seeding density, which was consistently maintained at 0.2 × 10⁶ viable cells/mL.

To clarify the number of cells used for BM-DCs generation, the **Methods** section, **Generation and culture of BM-DCs** subsection, has been revised as follows: **Lines** **134 - 137**: “ BM-DCs were generated by culture with either GM-CSF (Recombinant mouse GM-CSF, Biolegend, San Diego, USA, cat. 576308) alone or in conjunction with IL-4 (Recombinant mouse IL-4, R&D Systems, Minneapolis, USA, cat. 404-ML-050), starting from a **BM mononuclear cell suspension** of 0.2 x 10^6^ cells/mL in 6-well plates.”

3. Line 143: Non-adherent and loosely adherent cells were harvested by gently washing the well surface. What do they do with these cells? And the adherent ones? Do they try to immunophenotype the non-adherent and the adherent cells from GDCs and G4DCs cultures?

**Response:** The non-adherent and loosely adherent cells collected by gentle washing after bone marrow differentiation constituted the GDCs and G4DCs populations used in all subsequent experiments. These harvested cells were immunophenotyped under unstimulated conditions and following 24 h of LPS stimulation. The adherent cell fraction that remains attached to the culture wells was not harvested and was not used in subsequent experiments. Previous studies have shown that the firmly adherent fraction of murine GM-CSF-derived bone-marrow cultures is predominantly enriched in macrophage-like cells, whereas the non-adherent and loosely adherent fractions are commonly collected as BM-DCs populations (Wang et al., 2017). Ref: doi:10.3892/mmr.2017.7448.

The **Methods** section, **Generation and culture of BM-DCs** subsection, has been revised to clarify which cell fractions were harvested and analyzed. **lines 142 - 144:** “Harvested GDCs and G4DCs cells were used in subsequent flow cytometry and cytokine detection analysis. The firmly adherent fraction was discarded.”

4. Line 149: Do you try to measure the cytokines before LPS treatment to compare with after LPS treatment to compare the immature and mature DC phenotype? Take a look at these tho publications: <https://doi.org/10.1016/j.actatropica.2024.107426> and <https://doi.org/10.1016/j.imlet.2023.08.003>
Concerning cell markers, why don't they use CD1d and CD83?
--> marker for mature dendritic cells: CD83.

**Response:** Cytokine secretion was evaluated also before LPS stimulation and is noted in both phenotypic evaluation (Fig 2) and cytokine secretion (Fig 3) as the unstimulated condition. We have modified **line 148** to include the unstimulated control: "After 24 hours, the supernatant **of unstimulated and LPS-stimulated cells** was harvested for cytokine detection, while cells were used for phenotypic analysis.”

Regarding the phenotypic markers, our flow cytometry panel was selected to identify the murine BM-DCs cells and to evaluate their capacity for conventional protein-antigen priming of naive CD4⁺ T cells. CD1d is a specialised antigen-presenting molecule that primarily presents lipid and glycolipid antigens to natural killer T cells. In our study, the downstream *in vitro* model was designed to evaluate Th1 and Th2 associated responses to a protein antigen, rather than lipid-antigen presentation or NKT-cell activation.

We agree with the reviewer that CD83 is a recognized marker associated with activated and mature dendritic cells. However, CD83 was not included in the present study because in previous experiments we observed only low basal expression levels in both unstimulated and LPS-stimulated cells.

5. Line 156: Please include the gating strategy in a supplementary file. The number counts or log/linear numbers are missing in the files' histograms and plots. This is important for the readers.

**Response:** The number counts or log numbers are now added to all histograms and plots. We have also added the gating strategy as a supplementary figure (S4 Fig). In addition, the **Methods** section, **Cell surface molecule analysis** subsection, has been revised to also describe the gating steps and to include a reference to the corresponding supplementary figure (S4 Fig):

**Lines 174 - 178** : “After debris and doublet exclusion, DAPI-negative viable cells were selected followed by gating of the CD11c⁺ population. MHC-II and CD86 expression was assessed within the CD11c⁺ compartment as percentages and geometric median fluorescence intensity values (GeoMFI) of CD11c positive cells or CD11c+MHCII+/CD86+ cells. The gating strategy is shown in S4 Fig.”

6. Line 213: "In order to generate an optimal purity of DCs, as assessed by expression of the CD11c, the cell harvest period for GDCs was 8 days and 11 days for G4DCs". Just using CD11c? S1 Figure includes the gating strategy and puts the numbers (log/linear and counts).
Discussion: It is important to show the difference under Balbc mice DC-generation from other strains. This can bring different DC phenotypes.

**Response:** We used CD11c as the primary marker to identify the BM-DCs population because it is widely used to define murine dendritic cells generated in GM-CSF-based differentiation protocols. However, we agree that CD11c alone is not sufficient to fully characterize the phenotype or maturation status of the generated cells. Our objective was not to perform a detailed subset classification, but rather to use a focused phenotypic panel in which CD11c defined the BM-DCs population, MHC-II and CD86 and cytokine secretion were used to grossly evaluate the maturation status by expression of costimulatory molecules while antigen presentation was used to evaluate functionality.

To improve clarity, in the revised manuscript the gating strategy used for all flow cytometry experiments has now been added as supplementary S4 Fig. In addition, S1 Fig, which depicts the phenotypic analysis at different time points, was modified to include both G4DCs and GDCs and all plots axes log scale/events count were included.

While we do agree that the animal strain used for BM-DCs differentiation can significantly impact both differentiation and function, we have not currently explored using other mouse models.

A statement on this limitation has been added to the manuscript:

**Lines 427-429:** Also, as our study is limited to cells derived from BALB/c mice, further optimization will probably be required when using a different murine model.

If the authors used IA, it is important to mention it.

**Response:** AI was not used for the writing of this manuscript.

**Reviewer #2:**

The introduction of this manuscript is very well-written, setting up the background and highlighting the ongoing challenges regarding the heterogeneity of BM-DC differentiation protocols. However, some adjustments regarding the flow cytometry gating strategy, statistical documentation, and data presentation must be addressed to enhance the clarity and transparency of the manuscript.

**Response:** We thank the reviewer for their thoughtful comments and suggestions. We have revised the manuscript accordingly and hope that the changes adequately address all the points raised.

Because a new figure has been added, all subsequent figures have been renumbered accordingly. The line numbers cited in this response refer to the revised manuscript file labeled “Manuscript”.

1) In Figure 1, the authors evaluate the percentages of positive cells for individual markers (CD11c, MHC-II, and CD86). To ensure a rigorous immunological analysis, the correct approach should involve a sequential gating strategy: selecting single cells, gating on viable/live cells, identifying the CD11c+ population, and then assessing both the Median Fluorescence Intensity (MFI) and percentages for MHC-II and CD86 within that specific CD11c+ compartment.
• Please consider re-analyzing and presenting the data following this hierarchical structure and include the full gating strategy as a Supplementary Figure.

**Response:** While the recommended gating strategy was applied during the flow cytometry analysis to the CD11c+MHC+ and CD11c+CD86+ cells population, it was incorrectly mentioned in the manuscript. To clarify this aspect, we have added the gating strategy as a Supplementary Figure (S4 Fig). In addition, the **Methods** section, **Cell surface molecule analysis** subsection has been revised to also describe the gating steps and to include a reference to the corresponding supplementary figure (S4 Fig):

**174 - 178**: “After debris and doublet exclusion, DAPI-negative viable cells were selected followed by gating of the CD11c⁺ population. MHC-II and CD86 expression was assessed within the CD11c⁺ compartment as percentages and geometric median fluorescence intensity values (GeoMFI) of CD11c positive cells or CD11c+MHCII+/CD86+ cells. The gating strategy is shown in S4 Fig.”

• Ensure that the bar graphs showing percentages or MFIs are accompanied by representative flow cytometry dot plots/histograms.

**Response:** In the new manuscript, by addition of a new figure, Fig 1 became Fig 2. As such the figure has been revised and the legend was modified to include GeoMFI graphs, together with a representative flow cytometry histogram for each differentiation condition and analyzed marker.

• Additionally, Figure 1 currently lacks statistical comparisons; proper statistical testing between the relevant groups must be added to the figure

**Response:** The figure and its legend have been modified to include the statistical comparisons. Also, the **Methods** section, **Statistical analysis** subsection has been modified to include the normality test and the justification for the type of statistical test used for each dataset, including flow cytometry.

2) The justification for extending the culture of G4DCs to 10–11 days to achieve a higher and more homogeneous percentage of CD11c+ cells is reasonable for establishing a fair functional baseline and comparison. However, to strengthen this comparison, it would be highly interesting to see how GDCs behave if their culture is also extended for a few additional days. This would clarify whether the observed phenotypic differences are strictly cytokine-dependent or partially influenced by the time in culture (7–8 days vs. extended).

**Response:** In preliminary experiments we also evaluated an extended culture period of 10–11 days for GDCs. Under these conditions, the percentage of CD11c⁺ cells remained comparable to that observed after 7-8 days of culture but the expression of the co-stimulatory/activation markers MHC-II and CD86 was higher. Since GDCs already displayed a mature-like phenotype at day 7, further extending the culture could have additionally enhanced their baseline activation status, potentially due to prolonged exposure to growth factors. Therefore, we selected the 7-day culture protocol for GDCs in order to avoid further increasing the already elevated basal MHC-II and CD86 expression. The supplementary S1 Fig was modified to include an example of GDCs cultured for 10 days.

As this point was not mentioned in the original manuscript, we added the following statement to the **Results** section, subsection **Yield and phenotype of BM-DCs generated under specific cell culture conditions** at lines **238–240**: “Extending GDCs culture to 10–11 days did not increase the percentage of CD11c⁺ cells but further elevated basal MHC-II and CD86 expression. Therefore, GDCs were cultured for 7 to 8 days (S1B Fig).”

3) In the Methods section, please explicitly describe the decision-making workflow or criteria used to determine which specific statistical tests were applied to each experimental comparison (e.g., normality testing, pairing criteria, etc.)

**Response:** The **Methods** section, **Statistical analysis** subsection was modified accordingly by adding the following description of the statistical workflow:

**Lines** **213 - 224**: “Distribution normality was assessed group-wise using the Shapiro–Wilk test. Where normality assumptions were met the parametric student’s T-test was used to assess statistically significant differences between groups. T-tests were performed with Welch-Satterthwaite approximation to the degrees of freedom where unequal variances were assumed in unpaired samples. Data that deviated from a normal distribution were compared using non-parametric Wilcoxon signed-rank test (for paired samples) or Wilcoxon rank-sum test (for unpaired). Measurements were considered paired for unstimulated versus LPS stimulated cells within the same experiment under the same differentiation conditions and unpaired when comparing GDCs to G4DCs. Differences in cytokine secretion were assessed with parametric tests and non-parametric tests were applied on flow cytometry data. In all tests, p values were adjusted for multiple comparisons using the Benjamini-Hochberg procedure. Adjusted p values below 0.05 were considered statistically significant.”

4) To facilitate rapid interpretation, please add clear titles directly to the graphs and include visible legends identifying which group corresponds to each color, rather than relying solely on the text in the figure captions.

**Response:** Fig 2 was modified to include clear plot titles for each analysed marker box, as well as visible legends identifying the stimulation conditions corresponding to each colour. Fig 3 was also modified to include a colour legend. The supplementary figures were revised accordingly to follow the same specification.

5) The presentation of cytokine concentrations from ELISA supernatants on a logarithmic scale is unusual, as these values are typically reported as absolute integers (pg/mL or ng/mL). Please provide alternative literature justification where cytokine measurements from standard culture supernatants are plotted in log scale; otherwise, it is strongly recommended to display these concentrations using a standard linear scale.

**Response:** Cytokine concentrations were expressed in absolute units (pg/mL) however, the data were displayed on a logarithmic y-axis scale because LPS stimulation induced very high cytokine concentrations in some experimental conditions. When plotted on a standard linear scale these high values compressed the lower concentration range and obscured differences among the unstimulated and low-secretion conditions within the same cytokine plot which we found did not properly show differences in baseline cytokine levels between the two differentiation protocols. The logarithmic scale was therefore used solely to improve the visualization of all experimental groups within the same graph. Importantly, the logarithmic scale was used only for visualization, the statistical analyses were performed using the original non logarithmated data. To our knowledge, similar log10 scale plots are used in previous studies to represent data variation over several orders of magnitude. Examples of this kind of representation can be seen in Fig 2-4 <https://doi.org/10.1371/journal.pone.0086160> or <https://doi.org/10.1038/s41598-022-08618-x> Fig 4 and 5.
