# Supplementary material for: Phenotypic and functional patterns of murine bone marrow-derived dendritic cells influenced by in vitro differentiation conditions
Source: PLoS One. 2026 Aug 26;21(8):e0356806. doi: 10.1371/journal.pone.0356806 (PMC13513930; doi:10.1371/journal.pone.0356806)
Supplement: Peer review history

# Peer review: Decision Letter 0

**DOI:** 10.1371/journal.pone.0356806.r001

**Authors:** Francesco Bertolini

29 Jun 2026

Dear Dr. Ionescu,

Thank you for submitting your manuscript to PLOS ONE. After careful consideration, we feel that it has merit but does not fully meet PLOS ONE’s publication criteria as it currently stands. Therefore, we invite you to submit a revised version of the manuscript that addresses the points raised during the review process by bothe Reviewers.

Please submit your revised manuscript by Aug 28 2026 11:59PM. If you will need more time than this to complete your revisions, please reply to this message or contact the journal office at plosone@plos.org. When you're ready to submit your revision, log on to https://www.editorialmanager.com/pone/ and select the 'Submissions Needing Revision' folder to locate your manuscript file.

- A letter that responds to each point raised by the academic editor and reviewer(s). You should upload this letter as a separate file labeled 'Response to Reviewers'.
- A marked-up copy of your manuscript that highlights changes made to the original version. You should upload this as a separate file labeled 'Revised Manuscript with Track Changes'.
- An unmarked version of your revised paper without tracked changes. You should upload this as a separate file labeled 'Manuscript'.

If you would like to make changes to your financial disclosure, please include your updated statement in your cover letter. Guidelines for resubmitting your figure files are available below the reviewer comments at the end of this letter.

If applicable, we recommend that you deposit your laboratory protocols in protocols.io to enhance the reproducibility of your results. Protocols.io assigns your protocol its own identifier (DOI) so that it can be cited independently in the future. For instructions see: https://journals.plos.org/plosone/s/submission-guidelines#loc-laboratory-protocols. Additionally, PLOS ONE offers an option for publishing peer-reviewed Lab Protocol articles, which describe protocols hosted on protocols.io. Read more information on sharing protocols at https://plos.org/protocols?utm_medium=editorial-email&utm_source=authorletters&utm_campaign=protocols.

As the corresponding author, your ORCID iD is verified in the submission system and will appear in the published article. PLOS supports the use of ORCID, and we encourage all coauthors to register for an ORCID iD and use it as well. Please encourage your coauthors to verify their ORCID iD within the submission system before final acceptance, as unverified ORCID iDs will not appear in the published article. *Only*  the individual author can complete the verification step; PLOS staff *cannot*  verify ORCID iDs on behalf of authors.

We look forward to receiving your revised manuscript.

Kind regards,

Francesco Bertolini, MD, PhD

Academic Editor

PLOS One

Journal Requirements:

When submitting your revision, we need you to address these additional requirements.

1.Please ensure that your manuscript meets PLOS ONE's style requirements, including those for file naming. The PLOS ONE style templates can be found at

https://journals.plos.org/plosone/s/file?id=wjVg/PLOSOne_formatting_sample_main_body.pdf and

https://journals.plos.org/plosone/s/file?id=ba62/PLOSOne_formatting_sample_title_authors_affiliations.pdf

2. Thank you for stating the following financial disclosure:

“This work was supported by the Ministry of Research and Innovation, Romania, PCCDI - UEFISCDI, project number PN-III-P1-1.2-PCCDI-2017-0529, Contract 62PCCDI/2018 (acronym CONVAC) and through the Core Program within the National Research, Development and Innovation Plan 2022-2027, with the support of National Research Authority (ANC), project no. 23 44.”

Please state what role the funders took in the study.  If the funders had no role, please state: "The funders had no role in study design, data collection and analysis, decision to publish, or preparation of the manuscript."

If this statement is not correct you must amend it as needed.

Please include this amended Role of Funder statement in your cover letter; we will change the online submission form on your behalf.

3. If the reviewer comments include a recommendation to cite specific previously published works, please review and evaluate these publications to determine whether they are relevant and should be cited. There is no requirement to cite these works unless the editor has indicated otherwise.

[Note: HTML markup is below. Please do not edit.]

Reviewer's Responses to Questions

**Comments to the Author**

1. Is the manuscript technically sound, and do the data support the conclusions?

Reviewer #1: Yes

Reviewer #2: Yes

**********

2. Has the statistical analysis been performed appropriately and rigorously?

Reviewer #1: Yes

Reviewer #2: Yes

**********

3. Have the authors made all data underlying the findings in their manuscript fully available?

The PLOS Data policy

Reviewer #1: No

Reviewer #2: Yes

**********

4. Is the manuscript presented in an intelligible fashion and written in standard English?

Reviewer #1: Yes

Reviewer #2: Yes

**********

Reviewer #1: Dear authors,

The manuscript is interesting, and the obtained results show the impact of specific differentiation conditions on the

phenotypic and functional patterns of BM-DCs.

The Introduction section is well written, but the Results section is quite confusing. I suggest drawing an experimental design for better comprehension by the reader in the Generation and culture of BM-DCs, Methodology section.

Line 133: How many viable cells do you have conducting this procedure? I strongly suggest to include for other research.

Line 143: Non-adherent and loosely adherent cells were harvested by gently washing the well surface. What do they do with these cells? And the adherent ones? Do they try to immunophenotype the non-adherent and the adherent cells from GDCs and G4DCs cultures?

Line 149: Do you try to measure the cytokines before LPS treatment to compare with after LPS treatment to compare the immature and mature DC phenotype? Take a look at these tho publications: https://doi.org/10.1016/j.actatropica.2024.107426 and https://doi.org/10.1016/j.imlet.2023.08.003

Concerning cell markers, why don't they use CD1d and CD83?

--> marker for mature dendritic cells: CD83.

Line 156: Please include the gating strategy in a supplementary file. The number counts or log/linear numbers are missing in the files' histograms and plots. This is important for the readers.

Line 213: "In order to generate an optimal purity of DCs, as assessed by expression of the CD11c, the cell harvest period for GDCs was 8 days and 11 days for G4DCs". Just using CD11c? S1 Figure includes the gating strategy and puts the numbers (log/linear and counts).

Discussion: It is important to show the difference under Balbc mice DC-generation from other strains. This can bring different DC phenotypes.

If the authors used IA, it is important to mention it.

Reviewer #2: The introduction of this manuscript is very well-written, setting up the background and highlighting the ongoing challenges regarding the heterogeneity of BM-DC differentiation protocols. However, some adjustments regarding the flow cytometry gating strategy, statistical documentation, and data presentation must be addressed to enhance the clarity and transparency of the manuscript.

1) In Figure 1, the authors evaluate the percentages of positive cells for individual markers (CD11c, MHC-II, and CD86). To ensure a rigorous immunological analysis, the correct approach should involve a sequential gating strategy: selecting single cells, gating on viable/live cells, identifying the CD11c+ population, and then assessing both the Median Fluorescence Intensity (MFI) and percentages for MHC-II and CD86 within that specific CD11c+ compartment.

• Please consider re-analyzing and presenting the data following this hierarchical structure and include the full gating strategy as a Supplementary Figure.

• Ensure that the bar graphs showing percentages or MFIs are accompanied by representative flow cytometry dot plots/histograms.

• Additionally, Figure 1 currently lacks statistical comparisons; proper statistical testing between the relevant groups must be added to the figure

2) The justification for extending the culture of G4DCs to 10–11 days to achieve a higher and more homogeneous percentage of CD11c+ cells is reasonable for establishing a fair functional baseline and comparison. However, to strengthen this comparison, it would be highly interesting to see how GDCs behave if their culture is also extended for a few additional days. This would clarify whether the observed phenotypic differences are strictly cytokine-dependent or partially influenced by the time in culture (7–8 days vs. extended).

3) In the Methods section, please explicitly describe the decision-making workflow or criteria used to determine which specific statistical tests were applied to each experimental comparison (e.g., normality testing, pairing criteria, etc.)

4) To facilitate rapid interpretation, please add clear titles directly to the graphs and include visible legends identifying which group corresponds to each color, rather than relying solely on the text in the figure captions.

5) The presentation of cytokine concentrations from ELISA supernatants on a logarithmic scale is unusual, as these values are typically reported as absolute integers (pg/mL or ng/mL). Please provide alternative literature justification where cytokine measurements from standard culture supernatants are plotted in log scale; otherwise, it is strongly recommended to display these concentrations using a standard linear scale.

**********

what does this mean?). If published, this will include your full peer review and any attached files.

If you choose “no”, your identity will remain anonymous but your review may still be made public.

**Do you want your identity to be public for this peer review?**  For information about this choice, including consent withdrawal, please see our Privacy Policy

Reviewer #1: No

Reviewer #2: **Yes:** Jose R Pittaluga V

**********

[NOTE: If reviewer comments were submitted as an attachment file, they will be attached to this email and accessible via the submission site. Please log into your account, locate the manuscript record, and check for the action link "View Attachments". If this link does not appear, there are no attachment files.]

To ensure your figures meet our technical requirements, please review our figure guidelines: https://journals.plos.org/plosone/s/figures

You may also use PLOS’s free figure tool, NAAS, to help you prepare publication quality figures: https://journals.plos.org/plosone/s/figures#loc-tools-for-figure-preparation.

NAAS will assess whether your figures meet our technical requirements by comparing each figure against our figure specifications.

# Author comment: Author response to Decision Letter 1

**DOI:** 10.1371/journal.pone.0356806.r002

20 Jul 2026

As directed in the email received from the journal staff, a letter that responds to each point raised by the academic editor and reviewers was also upload as a separate file labeled 'Response to Reviewers.

Response to Reviewers

Journal Requirements:

When submitting your revision, we need you to address these additional requirements.

1.Please ensure that your manuscript meets PLOS ONE's style requirements, including those for file naming. The PLOS ONE style templates can be found at

https://journals.plos.org/plosone/s/file?id=wjVg/PLOSOne_formatting_sample_main_body.pdf and

https://journals.plos.org/plosone/s/file?id=ba62/PLOSOne_formatting_sample_title_authors_affiliations.pdf

Response: We have carefully reviewed the PLOS ONE formatting and style requirements, including the recommendations for file naming. The revised manuscript and all accompanying files have been formatted and named accordingly, following the provided PLOS ONE templates.

2. Thank you for stating the following financial disclosure:

“This work was supported by the Ministry of Research and Innovation, Romania, PCCDI - UEFISCDI, project number PN-III-P1-1.2-PCCDI-2017-0529, Contract 62PCCDI/2018 (acronym CONVAC) and through the Core Program within the National Research, Development and Innovation Plan 2022-2027, with the support of National Research Authority (ANC), project no. 23 44.”

Please state what role the funders took in the study. If the funders had no role, please state: "The funders had no role in study design, data collection and analysis, decision to publish, or preparation of the manuscript."

If this statement is not correct you must amend it as needed.

Please include this amended Role of Funder statement in your cover letter; we will change the online submission form on your behalf.

Response: We confirm that the funders had no role in the study. The following statement has been added to the cover letter:

“The funders had no role in study design, data collection and analysis, decision to publish, or preparation of the manuscript.”

3. If the reviewer comments include a recommendation to cite specific previously published works, please review and evaluate these publications to determine whether they are relevant and should be cited. There is no requirement to cite these works unless the editor has indicated otherwise.

Response: We reviewed the publications recommended by the reviewers and evaluated their relevance to the scope and findings of our study.

Reviewer #1:

Dear authors,

The manuscript is interesting, and the obtained results show the impact of specific differentiation conditions on the phenotypic and functional patterns of BM-DCs.

Response: We thank the reviewer for their thoughtful comments and suggestions. We have revised the manuscript accordingly and hope that the changes adequately address all the points raised.

Because a new figure has been added, all subsequent figures have been renumbered accordingly. The line numbers cited in this response refer to the revised manuscript file labeled “Manuscript”.

1. The Introduction section is well written, but the Results section is quite confusing. I suggest drawing an experimental design for better comprehension by the reader in the Generation and culture of BM-DCs, Methodology section.

Response: We added to the Methods section, Generation and culture of BM-DCs subsection, a drawing of the experimental design. The figure is marked in the manuscript in lines: 149 -150 and in figure 1 legend lines: 152-153 “Fig 1. Experimental design for BM-DCs differentiation and phenotypic and functional analyses. Created in BioRender. Ionescu, I. (2026) https://BioRender.com/vrb0luq” as required for licence compatibility between BioRender and PlosOne.

2. Line 133: How many viable cells do you have conducting this procedure? I strongly suggest to include for other research.

Response: For each BM-DCs batch, bone marrow mononuclear cells were obtained from one or more animals, depending on the requirements of the experiment and whether cells were also needed for additional experiments. The mean total cell yield was 42.8 ± 9.7 × 10⁶ cells per animal, with a viability of 92.8 ± 3.2%, corresponding to approximately 39.7 × 10⁶ viable cells per animal. Because the number of 6-well plates used varied among experiments, the total number of cells seeded per experiment also varied. Therefore to avoid confusion we reported the standardized initial seeding density, which was consistently maintained at 0.2 × 10⁶ viable cells/mL.

To clarify the number of cells used for BM-DCs generation, the Methods section, Generation and culture of BM-DCs subsection, has been revised as follows: Lines 134 - 137: “ BM-DCs were generated by culture with either GM-CSF (Recombinant mouse GM-CSF, Biolegend, San Diego, USA, cat. 576308) alone or in conjunction with IL-4 (Recombinant mouse IL-4, R&D Systems, Minneapolis, USA, cat. 404-ML-050), starting from a BM mononuclear cell suspension of 0.2 x 106 cells/mL in 6-well plates.”

3. Line 143: Non-adherent and loosely adherent cells were harvested by gently washing the well surface. What do they do with these cells? And the adherent ones? Do they try to immunophenotype the non-adherent and the adherent cells from GDCs and G4DCs cultures?

Response: The non-adherent and loosely adherent cells collected by gentle washing after bone marrow differentiation constituted the GDCs and G4DCs populations used in all subsequent experiments. These harvested cells were immunophenotyped under unstimulated conditions and following 24 h of LPS stimulation. The adherent cell fraction that remains attached to the culture wells was not harvested and was not used in subsequent experiments. Previous studies have shown that the firmly adherent fraction of murine GM-CSF-derived bone-marrow cultures is predominantly enriched in macrophage-like cells, whereas the non-adherent and loosely adherent fractions are commonly collected as BM-DCs populations (Wang et al., 2017). Ref: doi:10.3892/mmr.2017.7448.

The Methods section, Generation and culture of BM-DCs subsection, has been revised to clarify which cell fractions were harvested and analyzed. lines 142 - 144: “Harvested GDCs and G4DCs cells were used in subsequent flow cytometry and cytokine detection analysis. The firmly adherent fraction was discarded.”

4. Line 149: Do you try to measure the cytokines before LPS treatment to compare with after LPS treatment to compare the immature and mature DC phenotype? Take a look at these tho publications: https://doi.org/10.1016/j.actatropica.2024.107426 and https://doi.org/10.1016/j.imlet.2023.08.003

Concerning cell markers, why don't they use CD1d and CD83?

--> marker for mature dendritic cells: CD83.

Response: Cytokine secretion was evaluated also before LPS stimulation and is noted in both phenotypic evaluation (Fig 2) and cytokine secretion (Fig 3) as the unstimulated condition. We have modified line 148 to include the unstimulated control: "After 24 hours, the supernatant of unstimulated and LPS-stimulated cells was harvested for cytokine detection, while cells were used for phenotypic analysis.”

Regarding the phenotypic markers, our flow cytometry panel was selected to identify the murine BM-DCs cells and to evaluate their capacity for conventional protein-antigen priming of naive CD4⁺ T cells. CD1d is a specialised antigen-presenting molecule that primarily presents lipid and glycolipid antigens to natural killer T cells. In our study, the downstream in vitro model was designed to evaluate Th1 and Th2 associated responses to a protein antigen, rather than lipid-antigen presentation or NKT-cell activation.

We agree with the reviewer that CD83 is a recognized marker associated with activated and mature dendritic cells. However, CD83 was not included in the present study because in previous experiments we observed only low basal expression levels in both unstimulated and LPS-stimulated cells.

5. Line 156: Please include the gating strategy in a supplementary file. The number counts or log/linear numbers are missing in the files' histograms and plots. This is important for the readers.

Response: The number counts or log numbers are now added to all histograms and plots. We have also added the gating strategy as a supplementary figure (S4 Fig). In addition, the Methods section, Cell surface molecule analysis subsection, has been revised to also describe the gating steps and to include a reference to the corresponding supplementary figure (S4 Fig):

Lines 174 - 178 : “After debris and doublet exclusion, DAPI-negative viable cells were selected followed by gating of the CD11c⁺ population. MHC-II and CD86 expression was assessed within the CD11c⁺ compartment as percentages and geometric median fluorescence intensity values (GeoMFI) of CD11c positive cells or CD11c+MHCII+/CD86+ cells. The gating strategy is shown in S4 Fig.”

6. Line 213: "In order to generate an optimal purity of DCs, as assessed by expression of the CD11c, the cell harvest period for GDCs was 8 days and 11 days for G4DCs". Just using CD11c? S1 Figure includes the gating strategy and puts the numbers (log/linear and counts).

Discussion: It is important to show the difference under Balbc mice DC-generation from other strains. This can bring different DC phenotypes.

Response: We used CD11c as the primary marker to identify the BM-DCs population because it is widely used to define murine dendritic cells generated in GM-CSF-based differentiation protocols. However, we agree that CD11c alone is not sufficient to fully characterize the phenotype or maturation status of the generated cells. Our objective was not to perform a detailed subset classification, but rather to use a focused phenotypic panel in which CD11c defined the BM-DCs population, MHC-II and CD86 and cytokine secretion were used to grossly evaluate the maturation status by expression of costimulatory molecules while antigen presentation was used to evaluate functionality.

To improve clarity, in the revised manuscript the gating strategy used for all flow cytometry experiments has now been added as supplementary S4 Fig. In addition, S1 Fig, which depicts the phenotypic analysis at different time points, was modified to include both G4DCs and GDCs and all plots axes log scale/events count were included.

While we do agree that the animal strain used for BM-DCs differentiation can significantly impact both differentiation and function, we have not currently explored using other mouse models.

A statement on this limitation has been added to the manuscript:

Lines 427-429: Also, as our study is limited to cells derived from BALB/c mice, further optimization will probably be required when using a different murine model.

If the authors used IA, it is important to mention it.

Response: AI was not used for the writing of this manuscript.

Reviewer #2:

The introduction of this manuscript is very well-written, setting up the background and highlighting the ongoing challenges regarding the heterogeneity of BM-DC differentiation protocols. However, some adjustments regarding the flow cytometry gating strategy, statistical documentation, and data presentation must be addressed to enhance the clarity and transparency of the manuscript.

Response: We thank the reviewer for their thoughtful comments and suggestions. We have revised the manuscript accordingly and hope that the changes adequately address all the points raised.

Because a new figure has been added, all subsequent figures have been renumbered accordingly. The line numbers cited in this response refer to the revised manuscript file labeled “Manuscript”.

1) In Figure 1, the authors evaluate the percentages of positive cells for individual markers (CD11c, MHC-II, and CD86). To ensure a rigorous immunological analysis, the correct approach should involve a sequential gating strategy: selecting single cells, gating on viable/live cells, identifying the CD11c+ population, and then assessing both the Median Fluorescence Intensity (MFI) and percentages for MHC-II and CD86 within that specific CD11c+ compartment.

• Please consider re-analyzing and presenting the data following this hierarchical structure and include the full gating strategy as a Supplementary Figure.

Response: While the recommended gating strategy was applied during the flow cytometry analysis to the CD11c+MHC+ and CD11c+CD86+ cells population, it was incorrectly mentioned in the manuscript. To clarify this aspect, we have added the gating strategy as a Supplementary Figure (S4 Fig). In addition, the Methods section, Cell surface molecule analysis subsection has been revised to also describe the gating steps and to include a reference to the corresponding supplementary figure (S4 Fig):

174 - 178: “After debris and doublet exclusion, DAPI-negative viable cells were selected followed by gating of the CD11c⁺ population. MHC-II and CD86 expression was assessed within the CD11c⁺ compartment as percentages and geometric median fluorescence intensity values (GeoMFI) of CD11c positive cells or CD11c+MHCII+/CD86+ cells. The gating strategy is shown in S4 Fig.”

• Ensure that the bar graphs showing percentages or MFIs are accompanied by representative flow cytometry dot plots/histograms.

Response: In the new manuscript, by addition of a new figure, Fig 1 became Fig 2. As such the figure has been revised and the legend was modified to include GeoMFI graphs, together with a representative flow cytometry histogram for each differentiation condition and analyzed marker.

• Additionally, Figure 1 currently lacks statistical comparisons; proper statistical testing between the relevant groups must be added to the figure

Response: The figure and its legend have been modified to include the statistical comparisons. Also, the Methods section, Statistical analysis subsection has been modified to include the normality test and the justification for the type of statistical test used for each dataset, including flow cytometry.

2) The justification for extending the culture of G4DCs to 10–11 days to achieve a higher and more homogeneous percentage of CD11c+ cells is reasonable for establishing a fair functional baseline and comparison. However, to strengthen this comparison, it would be highly interesting to see how GDCs behave if their culture is also extended for a few additional days. This would clarify whether the observed phenotypic differences are strictly cytokine-dependent or partially influenced by the time in culture (7–8 days vs. extended).

Response: In preliminary experiments we also evaluated an extended culture period of 10–11 days for GDCs. Under these conditions, the percentage of CD11c⁺ cells remained comparable to that observed after 7-8 days of culture but the expression of the co-stimulatory/activation markers MHC-II and CD86 was higher. Since GDCs already displayed a mature-like phenotype at day 7, further extending the culture could have additionally enhanced their baseline activation status, potentially due to prolonged exposure to growth factors. Therefore, we selected the 7-day culture protocol for GDCs in order to avoid further increasing the already elevated basal MHC-II and CD86 expression. The supplementary S1 Fig was modified to include an example of GDCs cultured for 10 days.

As this point was not mentioned in the original manuscript, we added the following statement to the Results section, subsection Yield and phenotype of BM-DCs generated under specific cell culture conditions at lines 238–240: “Extending GDCs culture to 10–11 days did not increase the percentage of CD11c⁺ cells but further elevated basal MHC-II and CD86 expression. Therefore, GDCs were cultured for 7 to 8 days (S1B Fig).”

3) In the Methods section, please explicitly describe the decision-making workflow or criteria used to determine which specific statistical tests were applied to each experimental comparison (e.g., normality testing, pairing criteria, etc.)

Response: The Methods section, Statistical analysis subsection was modified accordingly by adding the following description of the statistical workflow:

Lines 213 - 224: “Distribution normality was assessed group-wise using the Shapiro–Wilk test. Where normality assumptions were

Attachment Submitted filename: Response to Reviewers.docx

# Peer review: Decision Letter 1

**DOI:** 10.1371/journal.pone.0356806.r003

**Authors:** Francesco Bertolini

6 Aug 2026

Phenotypic and functional patterns of murine bone marrow-derived dendritic cells influenced by in vitro differentiation conditions

PONE-D-26-24621R1

Dear Dr. Ionescu,

We’re pleased to inform you that your manuscript has been judged scientifically suitable for publication and will be formally accepted for publication once it meets all outstanding technical requirements.

Within one week, you’ll receive an e-mail detailing the required amendments. When these have been addressed, you’ll receive a formal acceptance letter and your manuscript will be scheduled for publication.

An invoice will be generated when your article is formally accepted. Please note, if your institution has a publishing partnership with PLOS and your article meets the relevant criteria, all or part of your publication costs will be covered. Please make sure your user information is up-to-date by logging into Editorial Manager at Editorial Manager® and clicking the ‘Update My Information' link at the top of the page. For questions related to billing, please contact billing support.

If your institution or institutions have a press office, please notify them about your upcoming paper to help maximize its impact. If they’ll be preparing press materials, please inform our press team as soon as possible -- no later than 48 hours after receiving the formal acceptance. Your manuscript will remain under strict press embargo until 2 pm Eastern Time on the date of publication. For more information, please contact onepress@plos.org.

Kind regards,

Francesco Bertolini, MD, PhD

Academic Editor

PLOS One

Additional Editor Comments (optional):

Reviewers' comments:

Reviewer's Responses to Questions

**Comments to the Author**

Reviewer #2: All comments have been addressed

**********

2. Is the manuscript technically sound, and do the data support the conclusions?

Reviewer #2: Yes

**********

3. Has the statistical analysis been performed appropriately and rigorously?

Reviewer #2: Yes

**********

4. Have the authors made all data underlying the findings in their manuscript fully available?

The PLOS Data policy

Reviewer #2: Yes

**********

5. Is the manuscript presented in an intelligible fashion and written in standard English?

Reviewer #2: Yes

**********

Reviewer #2: Authors have addressed all comments from reviewers, making a considerable improvement for the manuscript.

**********

what does this mean?). If published, this will include your full peer review and any attached files.

If you choose “no”, your identity will remain anonymous but your review may still be made public.

**Do you want your identity to be public for this peer review?**  For information about this choice, including consent withdrawal, please see our Privacy Policy

Reviewer #2: **Yes:** Jose R Pittaluga Villarreal

**********

# Editor report: Acceptance letter

**DOI:** 10.1371/journal.pone.0356806.r004

**Authors:** Francesco Bertolini

PONE-D-26-24621R1

PLOS One

Dear Dr. Ionescu,

I'm pleased to inform you that your manuscript has been deemed suitable for publication in PLOS One. Congratulations! Your manuscript is now being handed over to our production team.

At this stage, our production department will prepare your paper for publication. This includes ensuring the following:

* All references, tables, and figures are properly cited

* All relevant supporting information is included in the manuscript submission,

* There are no issues that prevent the paper from being properly typeset

You will receive further instructions from the production team, including instructions on how to review your proof when it is ready. Please keep in mind that we are working through a large volume of accepted articles, so please give us a few days to review your paper and let you know the next and final steps.

Lastly, if your institution or institutions have a press office, please let them know about your upcoming paper now to help maximize its impact. If they'll be preparing press materials, please inform our press team within the next 48 hours. Your manuscript will remain under strict press embargo until 2 pm Eastern Time on the date of publication. For more information, please contact onepress@plos.org.

You will receive an invoice from PLOS for your publication fee after your manuscript has reached the completed accept phase. If you receive an email requesting payment before acceptance or for any other service, this may be a phishing scheme. Learn how to identify phishing emails and protect your accounts at https://explore.plos.org/phishing.

If we can help with anything else, please email us at customercare@plos.org.

Thank you for submitting your work to PLOS One and supporting open access.

Kind regards,

PLOS ONE Editorial Office Staff

on behalf of

Dr. Francesco Bertolini

Academic Editor

PLOS One
